# Modulation of Mitochondrial Dynamics by the Angiotensin System in Dopaminergic Neurons and Microglia

**DOI:** 10.14336/AD.2024.0981

**Published:** 2025-10-22

**Authors:** Aloia Quijano, Ana I. Rodriguez-Perez, María Alicia Costa-Besada, Andrea Lopez-Lopez, María J. Guerra, Jose Luis Labandeira-Garcia, Rita Valenzuela

**Affiliations:** ^1^Research Center for Molecular Medicine and Chronic Diseases (CiMUS), Health Research Institute of Santiago de Compostela (IDIS), University of Santiago de Compostela, Santiago de Compostela, Spain.; ^2^Networking Research Center on Neurodegenerative Diseases (CIBERNED), Madrid, Spain.

**Keywords:** Angiotensin, fission, mitochondria, neuroinflammation, neurodegeneration, Parkinson

## Abstract

Renin-angiotensin system (RAS) dysfunctions have been associated to life-spam, and aging-related diseases, including neurodegenerative diseases, such as Parkinson’s disease, and the neuroinflammatory associated processes. Mitochondrial dysfunctions play a major role in aging-related diseases, including dopaminergic neurodegeneration and neuroinflammation. However, the mechanisms of RAS/mitochondria interactions remain to be clarified. In the present work, we studied the role of major RAS components in the mitochondrial dynamics in dopaminergic neurons and microglia using *in vitro* and *in vivo* models. In dopaminergic neurons, we observed that activation of the RAS pro-oxidative/pro-inflammatory axis (Angiotensin II/Angiotensin type-1 receptor, AT1/NADPH oxidase complex) produces a dysregulation of mitochondrial dynamics towards mitochondrial fission, via Drp1 phosphorylation at Ser616 and translocation to mitochondria. However, activation of the RAS antioxidative/anti-inflammatory axis, using Angiotensin 1-7, counteracts this effect. RAS components also modulated the microglial inflammatory response through mitochondrial dynamic changes. After interferon-γ-induced activation of human microglial cells, we observed increased mitochondrial fission and superoxide production that was inhibited by Angiotensin 1-7 treatment. Angiotensin 1-7 also inhibited mitochondrial metabolic changes induced by pro-inflammatory microglial activation. The role of RAS in mitochondrial dynamic changes was confirmed *in vivo* using the LPS-induced inflammation model in wild-type, AT1-KO, and AT2-KO mice. The effect of Angiotensin 1-7 is mediated by IL-10, specifically by decreasing the post-transcriptional phosphorylated Drp1 form, and translocation of STAT3 to mitochondria. Angiotensin 1-7, acting on mitochondrial Angiotensin 1-7 receptors (Mas/Mas related receptors), increased the phosphorylated form of STAT3 at Ser727, which is mediated by mitochondrial PKA activation. In conclusion, the present findings show the role of RAS components in modulation of mitochondrial dynamics and mitochondrial function, revealing the associated signaling pathways. The results lead to better understanding of the effects of RAS dysfunction in aging-related diseases, and particularly dopaminergic degeneration and neuroinflammation in Parkinson’s disease.

## INTRODUCTION

The renin-angiotensin system (RAS) was initially associated with blood pressure regulation. However, it is now known that there is local RAS in most organs, including the brain, that are involved in major cell and tissue functions [1]. Interestingly, numerous previous studies have shown that RAS dysfunction plays a major role in life-spam [2, 3], aging-related inflammatory processes (inflammaging; [4]) and aging-related diseases [5, 6], including neurodegenerative diseases such as Alzheimer disease [7, 8] and Parkinson’s disease (PD; [9]) and the neuroinflammatory associated processes. Mitochondrial dysfunctions play a major role in aging-related diseases, neuroinflammation and neurodegeneration [10, 11], including dopaminergic degeneration in PD [12, 13]. Consistent with the major role of RAS in aging and neurodegeneration, RAS activity modulates the mitochondrial function in aging and neurodegenerative processes [5, 9]. However, the mechanisms of RAS/mitochondria interactions remain to be clarified.

Mitochondrial dynamics (i.e. mitochondrial fusion and fission) plays a crucial role in mitochondrial function. The GTPase Dynamin-related protein 1 (Drp1) is the main effector molecule in mitochondrial fission. Drp1 mitochondrial recruitment and activity are regulated by several post-translational modifications such as phosphorylation, S-nitrosylation, ubiquitination, or O-GlcNAcylation [14]. The protein optic atrophy 1 (OPA1), acting on the mitochondrial inner membrane, plays a major role in mitochondrial fusion [15]. OPA1 exists in two forms: the membrane-anchored long form (L-OPA1) and short form (S-OPA1), which lacks the transmembrane domain and is generated by cleavage of L-OPA1 by the metallopeptidase OMA1 [16]. Mitochondrial inner-membrane fusion is regulated by the ratio of the two forms of OPA1, and excessive levels of S-OPA1 inhibit mitochondrial fusion [17].

RAS effects are mediated by two opposite arms/axes that counter-regulate each other [9, 18]. In dopaminergic neurons and glial cells, dysregulation of brain RAS towards the pro-oxidative/proinflammatory axis, constituted by Angiotensin II (Ang II) and its type-1 receptor (AT1), promotes oxidative stress by activation of the NADPH-oxidase complex and intracellular calcium raising, leading to neuroinflammation, neuronal degeneration, and disease progression [9, 19]. The effects of the RAS pro-oxidative axis are normally counteracted by the RAS antioxidative/anti-inflammatory axis, which consists of Ang II acting on its type 2 receptor (AT2) and the Ang II-derived peptide, angiotensin 1-7 (Ang 1-7) acting on Mas and Mas related receptors (MasR/MrgD/MrgE) [9, 20, 21]. Interestingly, in addition to RAS receptors located at the cell membrane, intracellular RAS receptors were observed in the mitochondria and nucleus of dopaminergic neurons and other cell types, which may counteract the pro-oxidative effects of the plasma membrane AT1 receptors [21-24].

Regarding PD, studies in different animal models have revealed the role of RAS dysregulation in dopaminergic degeneration and neuroinflammation [25-27], and recent studies in humans have shown that high levels AT1 gene expression identify the most vulnerable human dopaminergic neurons [28-30], and that treatment with AT1 blockers decreases the risk of PD development [31-33]. In microglia, the RAS has been shown to regulate the neuroinflammatory response [34, 35], and changes in mitochondrial dynamics have been involved in the microglial inflammatory response [36, 37]. However, the mechanistic link between RAS and mitochondrial dynamics in dopaminergic neurons and microglia has not been clarified. In the work reported here, we studied the role of major components of the pro-oxidative/proinflammatory RAS (Ang II/AT1) and anti-oxidative/anti-inflammatory RAS (Ang 1-7/Mas-MrgE receptors) in the mitochondrial dynamics in dopaminergic neurons and in the microglial inflammatory response, using *in vitro* and *in vivo* models.

## MATERIALS AND METHODS

### Experimental design

In the first set of experiments, we investigate the role of major components of the pro-oxidative/proinflammatory RAS (Ang II/AT1) and anti-oxidative/anti-inflammatory RAS (Ang 1-7/Mas-MrgE receptors) on the neuronal mitochondrial dynamics. We used N27 dopaminergic neurons treated with Ang II with or without Ang 1-7, as we previously observed that these cells are a suitable cellular model for studying the dopaminergic mitochondrial function [38]. We first studied the expression of the major mitochondrial dynamic effector molecules, Drp1 and OPA1, using RT quantitative PCR and Western blot (WB) analyses. Then, we analyzed the translocation of Drp1 to mitochondria by isolating cytosolic and mitochondrial fractions from neuron cultures after different experimental treatments. In cells labeled with the specific mitochondrial probe MitoTracker Deep Red, we analyzed the effect of treatments on mitochondrial morphology, calculating parameters of aspect ratio [AR; (major axis)/(minor axis)] and circularity [C; (4π·surface area)/(perimeter^2^)]. To study the role of NADPH-oxidase complex activation on the effects of Ang II on mitochondrial dynamics, we treated cultures with a potent NADPH-oxidase complex inhibitor, apocynin 30 min before Ang II treatment. Major results were confirmed in the substantia nigra of Sprague-Dawley rats intraventricularly injected with Ang II to ensure *in vivo* validation and provide better insight into mitochondrial dynamics in neurodegenerative conditions. We have previously shown that this model is suitable for detecting the effects of Ang II on the rat brain [39].

In a second set of experiments, we investigated the role of RAS components on the mitochondrial dynamics in the microglial inflammatory response using *in vitro* and *in vivo* models. We analyzed gene and protein expression of Drp1 and OPA1, translocation of Drp1 to mitochondria, and mitochondrial morphology in HMC3 human microglial cells treated with IFNγ, which mimic the inflammatory conditions observed in neurodegenerative diseases. In substantia nigra (SN) from an LPS inflammatory mouse model, we investigated the effect of deletion pro-oxidative (AT1; AT1KO mice) and anti-oxidative (AT2; AT2KO mice) RAS receptors on mitochondrial dynamics. LPS treatment induces a strong inflammatory response mimicking systemic and brain inflammation and oxidative stress.

A third set of experiments were designed to study functional effects of RAS on the metabolic shift and mitochondrial ROS production of microglial cells in response to inflammatory stimuli. Mitochondrial superoxide levels were measured using the specific fluorescent probe MitoSox and shifts in the metabolic state of microglial cells were measured by calculating the Glycolytic Proton Efflux Rate (glycoPER; rate of protons liberated to the medium during glycolysis) and real-time ATP production rate using a Seahorse Bioscience XF in HMC3 microglial cells treated with IFNγ.

A fourth set of experiments was conducted to identify potential mediators of the effects of RAS on mitochondrial dynamics. We first studied the possible link between Ang 1-7 and the transcription factor STAT3 in our model of inflammatory response using microglial cells, particularly, the non-canonical form of STAT3 phosphorylated at Serine 727 (pS727-STAT3), which is known as a mitochondrial function modulator. We analyzed STAT3 and pS727-STAT3 expression by WB in total cell lysate from HMC3 microglial cells treated with IFNγ with or without Ang 1-7. Next, we checked the expression of STAT3 and pS727-STAT3 at the mitochondrial fraction relative to cytosolic abundance. The effects of different treatments on STAT3 and pS727-STAT3 were analyzed by WB of isolated mitochondria and Immunofluorescence. Furthermore, to investigate the possible intramitochondrial phosphorylation of STAT3, isolated mitochondria from microglial cells were treated or not treated with Ang 1-7, Ang 1-7 receptor inhibitors (i.e., the MrgD/E antagonist D-Pro and Mas receptor antagonist A779), or the PKA inhibitor H-89, and analyzed by WB. PKA activity in isolated mitochondria was measured using a solid-phase enzyme-linked immuno-absorbent PKA activity assay.

We also studied whether IL-10 may mediate the effects of Ang 1-7 on mitochondrial dynamic changes and STAT3 Ser727 phosphorylation and translocation to mitochondria using WB and immunofluorescence in microglial cells treated or not treated with an IL-10 blocking antibody.

### Animal models

Tissue from substantia nigra of Ang II or saline-injected young adult Sprague-Dawley male rats and control or LPS treated wild type or AT1 receptor-deficient (AT1KO) or AT2 receptor-deficient (AT2KO) young adult C57BL-6 male mice were used for WB and RT-PCR studies. We used male rodents because we observed in previous studies that the effects of the lack of AT1 or AT2 receptors or Ang II administration are more marked and more easily identified in males [40]. Animal handling was conducted by Directive 2010/63/EU, European Council Directive 86/609/EEC, and the Spanish legislation (RD53/2013), in compliance with ARRIVE guidelines. Rodent experiments were approved by the corresponding committee at the University of Santiago de Compostela (USC) and Galician Government. Project title: Studies of new therapies in models of Parkinson's disease and other neurodegenerative diseases. Approval number: 15012/2021/012 (Last revision: 16 April 2021). Animals were housed at constant room temperature (RT) (21-22 °C) and 12-h light/dark cycle and 50% humidity with *ad libitum* access to food and water. Enrichment, including nesting materials and tunnels, was provided in all cages. Rodents were handled daily for at least five minutes over a 7-day acclimation period before the experimental procedures. The required sample size was estimated based on data from our previous studies to ensure adequate power to detect a pre-specified effect size and no animals were excluded from the statistical analysis, ensuring that all collected data were utilized in the results. A random number generator was used to assign each animal to one of the experimental groups, ensuring an equal probability of assignment and preventing selection bias. Finally, the investigator was blinded to the group allocation throughout the experiment to prevent bias in the data collection and analysis processes.

### Intraventricular angiotensin II injections

Young adult male (8-week-old) Sprague-Dawley rats (N=7), weighing between 250-300 g, were anesthetized with a pharmacological combination including ketamine (50 mg/kg, Ketamidor, CN:580393.7, Richter Pharma) and medetomidine (0.4 mg/kg, Domtor, CN:570686.3, Ecuphar) before stereotaxic surgery (Kopf Instruments) to inject the Ang II peptide into the third ventricle [41, 42]. The dental bar was set at 0 and the following midline coordinates were used from bregma: -0.8mm anteroposterior and -6.5mm ventral to the cranial surface. The skull was then trephined and a concentration of 5µg of Ang II in a volume of 3 µl of sterile saline solution was injected (at a rate of 0.5 µl/min) using a 10 µl Hamilton syringe attached to a monitored injector (Stoeling). Controls (N=7) corresponding to this treatment group were injected with the same volume of saline. Sixteen hours after injection, the animals were anesthetized as above, decapitated and the substantia nigra dissected for the study of mitochondrial dynamic markers using the WB and PCR.

### Mouse LPS injection

Male young adult (8-week-old) mice wild-type (N=6, WT, C57BL-6 mice from Charles River, L’Arbresle, France) or homozygous AT1a deficient mice (8-week-old) (N=5, AT1 KO; Jackson Laboratory, Bar Harbor, ME, USA) or homozygous AT2 deficient mice (8-week-old) (N=6, AT2 KO, gift of Dr. Daniel Henrion) were diary intraperitoneally injected or not (WT controls) with Lipopolysaccharides from Escherichia coli (LPS) (L2880; Sigma, St. Louis, MO, USA); 2,5mg/mL. The drug was administered at a dose of 5 mg/kg. Six hours later, mice were anesthetized, sacrificed, and the brains quickly removed from the skull and placed in a mouse stainless-steel brain matrix (51388, Stoelting Co., Wood Dale, IL, USA). The ventral mesencephalon was dissected on a pre-cooled glass plate, and quickly frozen at -80ºC and stored. Tissue samples were processed for RNA and protein analysis by RT-PCR and WB.

### Cell cultures and treatments

The N27 dopaminergic neuron cell line (SCC048, Millipore) was cultured in RPMI 1640 medium supplemented with 10% FBS, 2 mM L-glutamine (Sigma), 100 U/ml penicillin, and 100 μg/ml streptomycin. The HMC3 human fetal brain-derived primary microglial cells (ATCC Cat# CRL-3304) were cultured in DMEM medium (Sigma) with 2 mM L-Glutamine (Sigma), 100 U/ml penicillin and 100-μg/ml streptomycin. Cultures were maintained at 37 °C and 5% CO_2_ in a humidified incubator. Both cell lines were recently tested for mycoplasma contamination using PCR and were confirmed to be free of contamination.

Rat N27 dopaminergic neurons were treated with Angiotensin II (Ang II; 1 μM, A9525, Sigma) for 6 h. HMC3 human microglial cells were treated with interferon-gamma (IFNγ; 100ng/ml, PHC4031, Gibco) for 24 h. to induce microglial activation. To test the effect of activation of the RAS protective axis, angiotensin 1-7 (Ang 1-7; 1µM, A9202 Sigma) was added to the cultures 30 min before the treatments (IFNγ or Ang II). Control cellular group did not receive the experimental treatment to allow for comparison against experimental group. To study the role of the NADPH-oxidase complex, cultures were treated with a potent inhibitor of NADPH-oxidase complex, apocynin (100µM, 11458653 ThermoScientific) was added to the cultures 30 min before Ang II treatment.

To study the role of IL-10, HMC3 cells were incubated in a starvation medium (DMEM and antibiotics) and seeded in 12-well plates for 48 h (25 x 10^4^ cells/well), and then treated with a IL-10 blocker. Blockage of IL-10 was performed using an excess of anti-hIL-10 antibody (5μg/mL; R&D systems Cat# MAB2171) 30 min before treatments as previously described [43]. We tested the effect of IL-10 blockage on Ang 1-7 + IFNγ treated cells. After 24 h, cells were washed and processed for WB or RT-PCR.

### Western blot analysis

SN homogenates from rat and mouse models and cell culture total/cytosolic fraction or mitochondrial fraction were lysed in RIPA buffer containing PMSF (Sigma), phosphatase inhibitor cocktail (Sigma), and protease inhibitor cocktail (Sigma). Cellular and tissue lysates were centrifuged, and total proteins were quantified using the Pierce BCA Protein Assay Kit (Thermo Scientific). Furthermore, 10 µg of isolated mitochondria from HMC3 microglial cells were treated or not treated with Ang 1-7 (1 µM; 10 min), with or without preincubation (5 min) with different inhibitors: Ang 1-7 receptors inhibitors (the MrgD/E antagonist D-Proline; D-Pro, 5 µM; and the Mas receptor antagonist A779; 1 µM) or the PKA inhibitor H-89 (10 µM; 371963, Millipore). Then mitochondria were lysed in RIPA buffer containing PMSF (Sigma), phosphatase inhibitor cocktail (Sigma), and protease inhibitor cocktail (Sigma). An equal amount of protein lysates was separated on a 10 % Bis-Tris polyacrylamide gel and transferred to nitrocellulose membranes. Membranes were incubated overnight at 4 °C with primary antibodies against DRP1 protein (1:2000; BD Cat# BD611112), Phospho-DRP1 S616 (1:1000; Cell Signaling Technology Cat# 4494), OPA-1 (1:1000; BD Cat# BD612606), STAT3 (1:1000; Abcam Cat# ab68153) or STAT3 (phospho S727) (1:1000; Abcam Cat# ab32143). Membranes were reincubated with loading controls: anti-α-tubulin (1:50000; Sigma-Aldrich Cat# T5168), GAPDH (1:25000; Sigma-Aldrich Cat# G9545), or VDAC/porin (1:1.000; Sigma-Aldrich Cat# V2139).

Horseradish peroxidase (HRP)-conjugated secondary antibodies were used: goat anti-rabbit-HRP and goat anti-mouse-HRP (1:2500; Santa Cruz Biotechnology Cat# sc-2030). Detection of antibodies bounded was performed with an Immun-Star HRP Chemiluminescent Kit (Bio-Rad; 170-5044) and visualized with a chemiluminescence detection system (Bio-Rad; Molecular Imager ChemiDoc XRS System). Data were then expressed relative to the value obtained for the control to counteract possible variability among batches. As the mitochondrial fission main protein, Drp1 is regulated by several post-translational modifications, the ratio between activated phosphorylation of serine residue S616 of Drp1(p-Drp1) and total Drp1 was calculated as an indicative of DRP1-mediated mitochondrial fission [44].

### Translocation of Drp1 to mitochondria

Changes in the intracellular location of Drp1 were assessed by measuring protein levels at isolated cytosolic and mitochondrial fractions from cell culture after experimental treatments. Mitochondria were isolated from dopaminergic N27 and microglial HMC3 cells seeded in 150 mm culture dishes at a concentration of 10^7^ cells/plate using a commercial mitochondrial isolation kit for cultured cells (89874, Thermo Scientific) following manufacturer’s instructions. This protocol relies on differential centrifugation to separate mitochondrial and cytosolic fractions. Before centrifugation, dounce homogenization was performed to increase the amount of mitochondrial fraction. Subcellular fractionation products were analyzed by WB with an anti-Drp1 antibody using GAPDH as a housekeeping protein. The ratio of cytosolic and mitochondrial Drp1 expression (Cyt/Mit) was calculated to quantify Drp1 translocation rate changes to mitochondria [45].

### RNA extraction and real-time quantitative polymerase chain reaction

Following the manufacturer's protocol, the total RNA from rat and mouse SN and HMC3 or N27 cell lysates were extracted with TRIzol (Invitrogen, Paisley, UK). Total RNA (2 μg) was reversed transcribed to complementary DNA (cDNA) using nucleoside triphosphate containing deoxyribose, random primers and Moloney murine leukemia virus (M-MLV; Invitrogen, Thermo Fisher Scientific; 200U) reverse transcriptase. Subsequently, the RT-PCR analysis was performed with a QuantStudio 3 platform (Applied Biosystems, Foster City, CA, USA), the EvaGreen qPCR MasterMix (Applied Biological Materials Inc., Vancouver, Canada), and primer sequences indicated below were used to examine the relative levels of Drp1 and OPA1. β-Actin was used as a housekeeping gene and was amplified in parallel with the genes of interest. Forward (F) and reverse (R) primers were designed for each gene by using NCBI Primer-BLAST. We used the comparative cycle threshold values (cycle threshold (Ct)) method (2^- ΔΔCt^) to examine the relative messenger RNA (mRNA) expression. A normalized value was obtained by subtracting the Ct of β-actin from the Ct of interest (ΔCt). As it is uncommon to use ΔCt as a relative expression data due to this logarithmic characteristic, the 2^- ΔΔCt^ parameter was used to express the relative expression data. The list of Primers used in this study is shown in Table 1.

**Table 1:** List of Primers Used in This Study.

Primers	Sequences
**Human IL-1B -F**	5´-GATGGCTTATTACAGTGGCAA-3´
**Human IL-1B -R**	5´-GAGATTCGTAGCTGGATGC-3´
**Human IL-6 -F**	5´-AAATTCGGTACATCCTCGAC-3´
**Human IL-6 -R**	5´-CAGGCAAGTCTCCTCATTG-3´
**Human Drp1 -F**	5´-ATCCAGCTGCCTCAAATCGT-3´
**Human Drp1 -R**	5´-TCTGCTTCCACCCCATTTTCT-3´
**Human OPA1 -F**	5´-AGTTAGCACCAGACTTTGAC-3´
**Human OPA1 -R**	5´-GCAGACCCTTTCTAAAATGC-3´
**Rat Drp1-F**	5´-ACCTGACGCTTGTGGATT-3´
**Rat Drp1-R**	5´-GTGACAGCAAGGATAATGGAAT-3´
**Rat OPA1-F**	5´-GAAGTCTGCCAATCCTTA-3´
**Rat OPA1-R**	5´-AGGTTAGTTAGAGAAGAGAATT-3´
**Mouse IL-1B-F**	5´-GCTATGGCAACTGTTCCTGA -3´
**Mouse IL-1B-R**	5´-TGATGTGCTGCTGCGAGA-3´
**Mouse Drp1-F**	5´-ACCTGACACTTGTGGATT-3´
**Mouse Drp1-R**	5´-GGCGAGGATAATGGAATTG-3´
**Mouse OPA1-F**	5´-CCTGTGAAGTCTGCCAAT-3´
**Mouse OPA1-R**	5´-AAGGTGAGTTAGAGAAGAGAAC-3´

Drp1: Dynamin-related protein 1, F: Forward, IL-1B: interleukin 10, IL-6: interleukin 6, OPA1: optic atrophy 1, R: Reverse.

### Assessment of mitochondrial morphology

Mitochondrial morphology was assessed in N27 and HMC3 cells labelled with 200 nM of MitoTracker Deep Red (MTDR; M22426, Invitrogen) and the DNA-binding dye Hoechst 33 342 (Sigma; 10 μg/ml) using a Leica SP5 confocal microscopy (AOBS-SP5X; Leica Microsystems Heidelberg GmbH, Mannheim, Germany). Thirty to forty cells per group were analyzed with Fiji/ImageJ software (National Institutes of Health, Bethesda, MD) following a previously described protocol [46] with some modifications. Briefly, the images were processed using several filters to increase the signal-to-noise ratio and avoid losing information after binarization and thresholding. Once the images were binarized and thresholding, particle analysis was done to generate and calculate characteristics of mitochondrial morphology like aspect ratio [AR; (major axis)/(minor axis)] and circularity [C; (4π·surface area)/(perimeter^2^)].

### Metabolic rate measurements: glycolytic proton efflux rate and ATP production rate

Shifts in the metabolic state of microglial cells were measured by calculating the Glycolytic Proton Efflux Rate (glycoPER; rate of protons liberated to the medium during glycolysis) and real-time ATP production rate using a Seahorse Bioscience XF Extracellular Flux Analyzer (Seahorse Bioscience, North Billerica, MA). GlycoPER (pmol/min/μg of protein), which represents the lactate production rate, was calculated for basal glycolysis and compensatory glycolysis (ATP production blocked with rotenone and antimycin). Finally, the addition of 2-deoxy-Dglucose (2-DG) confirmed the amount of acidification caused by glycolysis. HMC3 cells (15 × 10^3^ cells/well) were seeded in 96-well plates and grown for 48 h. After treatments, cells were washed and incubated for 1 h at 37 °C in a non-CO_2_ incubator with assay medium (XF DMEM base medium supplemented with 2 mM glutamine, 10 mM glucose, and 1 mM sodium pyruvate, pH 7.4). A glycolytic rate assay was performed where rotenone and antimycin A (0.5 μM) and 2-DG (50 mM) were added sequentially. Real-time ATP rate measures simultaneously basal ATP production rates from mitochondrial respiration and glycolysis. Seahorse XF Real-Time ATP Rate Assay Kit employs a sequential injection of Oligomycin (1.5 μM) and Rotenone/Antimycin A (0.5 μM). GlycoPER and real-time ATP rate were calculated at different parameter values by Wave Desktop 2.6.1. Data were normalized with total μg of protein calculated with Pierce BCA Protein Assay Kit (Thermo Scientific).

### Mitochondrial superoxide production

Mitochondrial superoxide levels were measured using the specific fluorescent probe MitoSox (5μM; M36008, ThermoFisher). HMC3 cells were seeded in 12-well plates (10^5^ cells/well) covered with a glass coverslip. After treatments, the cells were incubated with MitoSox at 5μM for 30 min at 37ºC. Cells were fixed and analyzed by confocal laser microscopy (AOBSSP5X; Leica Microsystems Heidelberg GmbH, Mannheim, Germany) performing sequential scan to avoid any potential overlap with the LAS AF software (Leica Microsystems GmbH). Fluorescence intensity quantification was done with the open-source image processing package based on ImageJ software (NIH, Bethesda, MD, USA, rsb.info.nih.gov/ij/) calculating corrected total cell fluorescent index (CTCF) = Integrated density- (Area of cell x Mean fluorescence of background).

### Immunofluorescence labelling

Cells were grown on glass coverslips and treated with IFNγ for 24 h with or without Ang 1-7 and with or without IL-10 blocker, 30 min before the IFNγ treatment. Cells were incubated overnight at 4ºC with STAT3 (1:100; Abcam Cat# ab68153) or STAT3 (phospho S727) (1:100; Abcam Cat# ab32143) and TOMM20 (1:100; Santa Cruz Biotechnology Cat# sc-17764) as a mitochondrial marker. Primary antibodies were diluted in DPBS containing 1 % BSA and 2 % normal donkey serum. Then, the following fluorescent secondary antibodies were incubated for 2 h at RT: Alexa Fluor 568-conjugated donkey anti-rabbit IgG (1:200; Thermo Fisher Scientific Cat# A10042) or Alexa Fluor 488-conjugated donkey anti-mouse IgG (1:200; Thermo Fisher Scientific Cat# A-21202). The DNA-binding dye Hoechst 33 342 (Sigma; 10 μg/ml), 30 min at RT, was used to mark cell nuclei. Mounting was performed with Immumount (Thermo-Shandon). Co-localization of markers was confirmed by confocal laser microscopy (AOBS-SP5X; Leica Microsystems Heidelberg GmbH, Mannheim, Germany) with the LAS AF software (Leica Microsystems GmbH). The specificity of the antibodies used for immunostaining of STAT3 and STAT3 (phospho S727) was established by the commercial supplier, Abcam, with different advanced validation strategies, including KO validation. Moreover, these primary antibodies were also validated in previous studies [47]. In the present study, we confirmed specificity of STAT3 and phospho S727-STAT3 fluorescence signals by performing secondary antibody control in immunocytochemistry assays (Supplementary Fig.1)

### PKA activity assay

Intramitochondrial PKA activity was measured using a commercial PKA activity assay kit (ab139435, Abcam) based on a solid phase enzyme-linked immuno-absorbent assay (ELISA) with a specific synthetic peptide as a substrate for PKA and a polyclonal antibody that recognizes the phosphorylated form of the substrate. Isolated mitochondria from HMC3 cells were treated or not with Ang 1-7 (1 µM) for 10 min, with or without 5 min-preincubation with Ang 1-7 receptors inhibitors (see above). The PKA inhibitor H-89 (10 µM; 371963, Millipore) was used as an internal control of the assay. Then, 30 µl containing 100 ng of isolated mitochondria were added to a precoated 96-well plate, and the kinase reaction was started with the addition of ATP solution for 90 minutes at 30ºC. PKA activity of the samples was finally measured by absorbance (450 nm) using an Infinite 200 PRO multiwell plate reader (TECAN), after incubation with PKA substrate antibody for 60 min and HRP-secondary antibody for another 30 min.

### Statistical analysis

All statistical analyses were conducted using SigmaPlot 11.0 (Systat Software, Inc., CA, USA). Datasets were first tested for normality using the Kolmogorov-Smirnov test. If the dataset met the assumptions of normality and homoscedasticity (assessed by Levene's test), parametric tests were applied. Specifically, a Student’s t-test was used for comparing two independent groups, while one-way ANOVA followed by post-hoc comparisons using the Student-Newman-Keuls method was employed for more than two groups. In cases where the assumption of normality was violated, non-parametric tests were conducted: the Mann-Whitney U test for two-group comparisons and the Kruskal-Wallis test for comparisons across multiple groups. Post-hoc pairwise comparisons for non-parametric data were performed using Dunn's Method. Missing data occurred at random and statistical analysis was performed with the available data. Differences between groups were considered statistically significant at p < 0.05. The choice of the Student-Newman-Keuls method for post-hoc testing was based on its ability to control the familywise error rate in balanced designs, while Dunn's Method was selected for non-parametric data to account for multiple comparisons in an unbiased manner.

**Figure 1.**
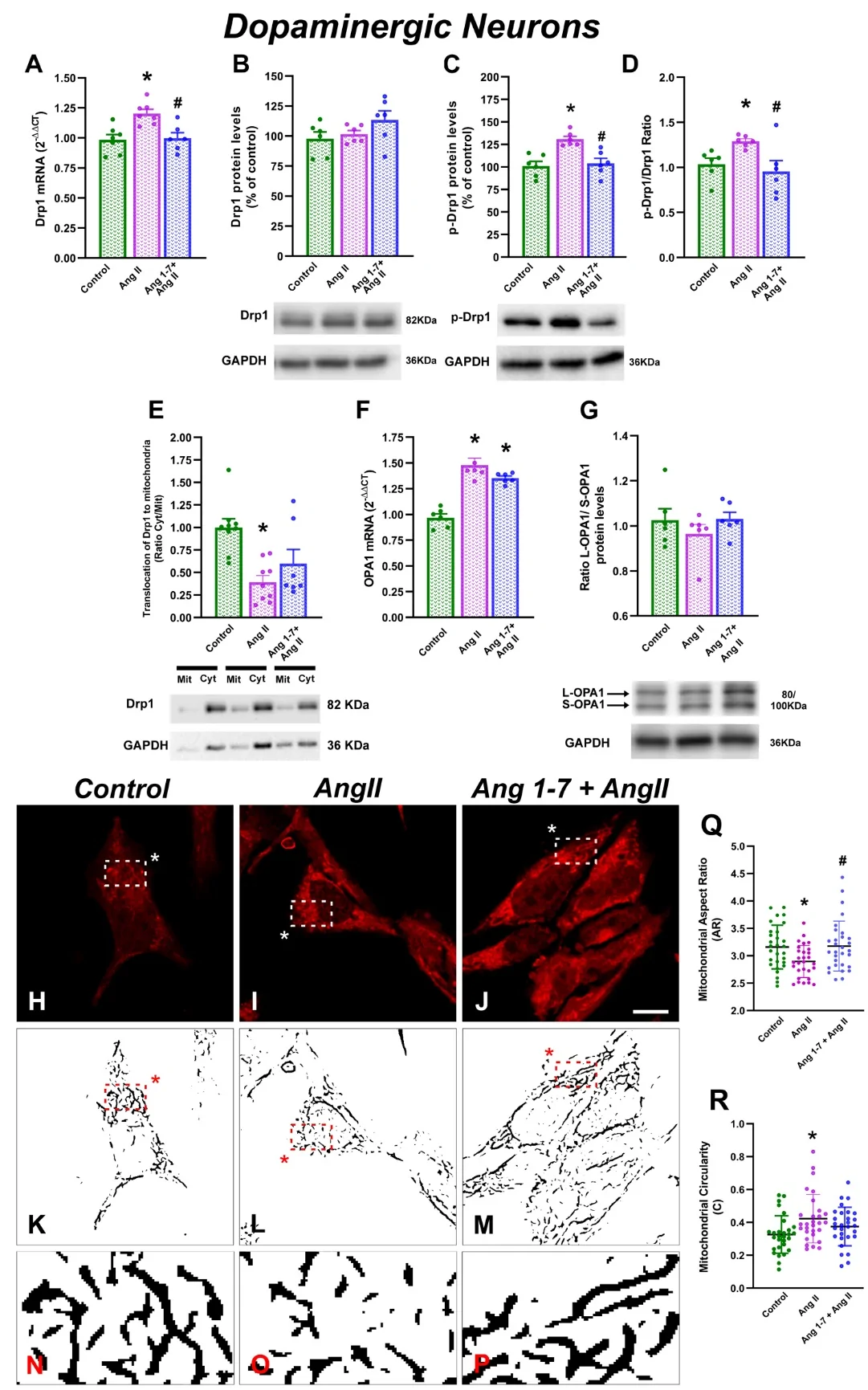
**Regulation of mitochondrial dynamics by angiotensin in dopaminergic neurons**. (A, C) Levels of Drp1 mRNA (N=6-7) and the phosphorylated form of Drp1 at Serine 616 (p-Drp1) (N=6) were significantly increased after Ang II treatment, but not in cells pre-treated with Ang 1-7 (B) Protein Drp1 levels were not affected by Ang II or Ang 1-7 treatment (D) Ang II increased the p-Drp1/Drp1 ratio, which was inhibited by Ang 1-7 (N=6) (E) Ang II treatment increased the translocation of Drp1 from the cytosol to mitochondria, as indicated by the decrease in the ratio between cytosolic and mitochondrial protein levels, which was inhibited by Ang 1-7 (N=7-9) (F) Fusion protein OPA1 mRNA was increased by Ang II treatment and not significantly inhibited by Ang 1-7 (N=6) (G) No significant change was observed in the ratio of the two forms of OPA1 (L-OPA1/S-OPA1) protein with treatments (N=6) (H-P) Mitochondria were stained with MTDR (H-J), and images were processed and analysed for area, perimeter, major, and minor axis using Fiji/ImageJ software to calculate the mitochondrial shape descriptors aspect ratio [AR] and circularity [C] (N-P boxed area magnified). (**Q-R**) Ang II treatment produced a significant change in mitochondrial morphology towards higher fragmentation as shown by a decrease in AR, and an increase in circularity. Ang 1-7 pre-treatment produced more elongated mitochondrial morphology compared with the Ang II treatment alone. Ten pictures were analyzed for each treatment (N=29-32 cells per group). Data are representative of three independent experiments and presented as mean ± SEM. *p < 0.05; One Way ANOVA with Student-Newman-Keuls Method post hoc test: A (P =0.002), B (P =0.167), C (P < 0.001), F (P < 0.001), G (P < 0.001); Kruskal-Wallis One Way Analysis of Variance on Ranks; D (P < 0.001), E (P = 0.007), Q (P =0.016), R (P =0.017). Abbreviations: Ang II, Angiotensin II; Ang 1-7, Angiotensin 1-7; Drp1, Dynamin-related protein 1; L-OPA1, long optic atrophy 1, S-OPA1, short optic atrophy 1, GAPDH, Glyceraldehyde-3-phosphate dehydrogenase; Mit, Mitochondrial fraction; Cyt, Cytosolic fraction, AR, aspect ratio; C, circularity.

## RESULTS

### Mitochondrial fission is induced by Ang II, via NADPH-oxidase activation, and inhibited by Ang 1-7 in dopaminergic neurons

We first investigated whether Ang II affects the mitochondrial dynamics of dopaminergic neurons. After 6 hours of treatment with 100 nM Ang II, levels of mitochondrial fission Drp1 mRNA were increased relative to control cells, although we did not detect changes in Drp1 protein expression (Fig. 1A, B). However, Ang II treatment produced an increase in Drp1 phosphorylation at Ser616 (p-Drp1), and consequently in p-Drp1/Drp1ratio. This phosphorylation activates Drp1 and induces mitochondrial fragmentation [45] (Fig. 1C, D). Then, we studied changes in translocation levels of Drp1 protein to mitochondria, as it is known that the mitochondrial fission process requires an additional step of translocation of Drp1 from its location at the cytosol, associated with microtubules, to mitochondria where it binds to the outer membrane at fission points [48]. Our results show that Ang II produced an increase in translocation of Drp1, as shown by the significant decrease of cytosol/mitochondria ratio of Drp1 protein expression in Ang II-treated cells compared with non-treated cells (Fig. 1E). All the Ang II-dependent effects on phosphorylation and translocation of Drp1 did not occur when we preincubated the cells with the peptide Angiotensin 1-7 (Ang 1-7), 30 min before Ang II treatment (Fig. 1A-E), confirming a role of the RAS in modulation of dopaminergic mitochondrial dynamics.

We also studied the mitochondrial fusion process by analysing the expression of the main mediator fusion protein, OPA1. We observed an increase in mRNA OPA1 expression after administration of Ang II alone or Ang II plus Ang 1-7, but no significant change was observed at the protein level. Ang II administration induced a non-significant decrease in the ratio between the two forms of OPA1 (L-OPA1/S-OPA1), which was not observed in the presence of Ang 1-7 pretreatment (Fig. 1F, G). These results suggest that RAS induces major effects on the mitochondrial dynamics of dopaminergic cells through Drp1 phosphorylation at Ser616 and translocation to mitochondria. The higher expression of OPA1 mRNA may represent a mechanism to compensate for the dynamic disequilibrium related to the Ang II-induced increase in mitochondrial fission.

The effect of Ang II treatment on mitochondrial morphology was then evaluated by labeling mitochondria with the specific probe, MitoTracker Deep Red (MTDR), and by analyzing mitochondria patterns with confocal microscopy. Dopaminergic N27 cells treated with Ang II for 6 hours (Fig. 1I, L, O) showed shorter and rounder mitochondrial shapes compared with non-treated cells (Fig. 1H, K, N). Aspect ratio (AR) and circularity values were calculated from confocal images using Fiji Image J software. Ang II treatment produced a significant change in mitochondrial morphology, as revealed by the decrease in AR (Fig. 1Q), along with an increase in circularity factor (Fig. 1R). These changes were not produced with Ang 1-7 preincubation, supporting the above-mentioned results (Fig. 1J, M, P).

**Figure 2.**
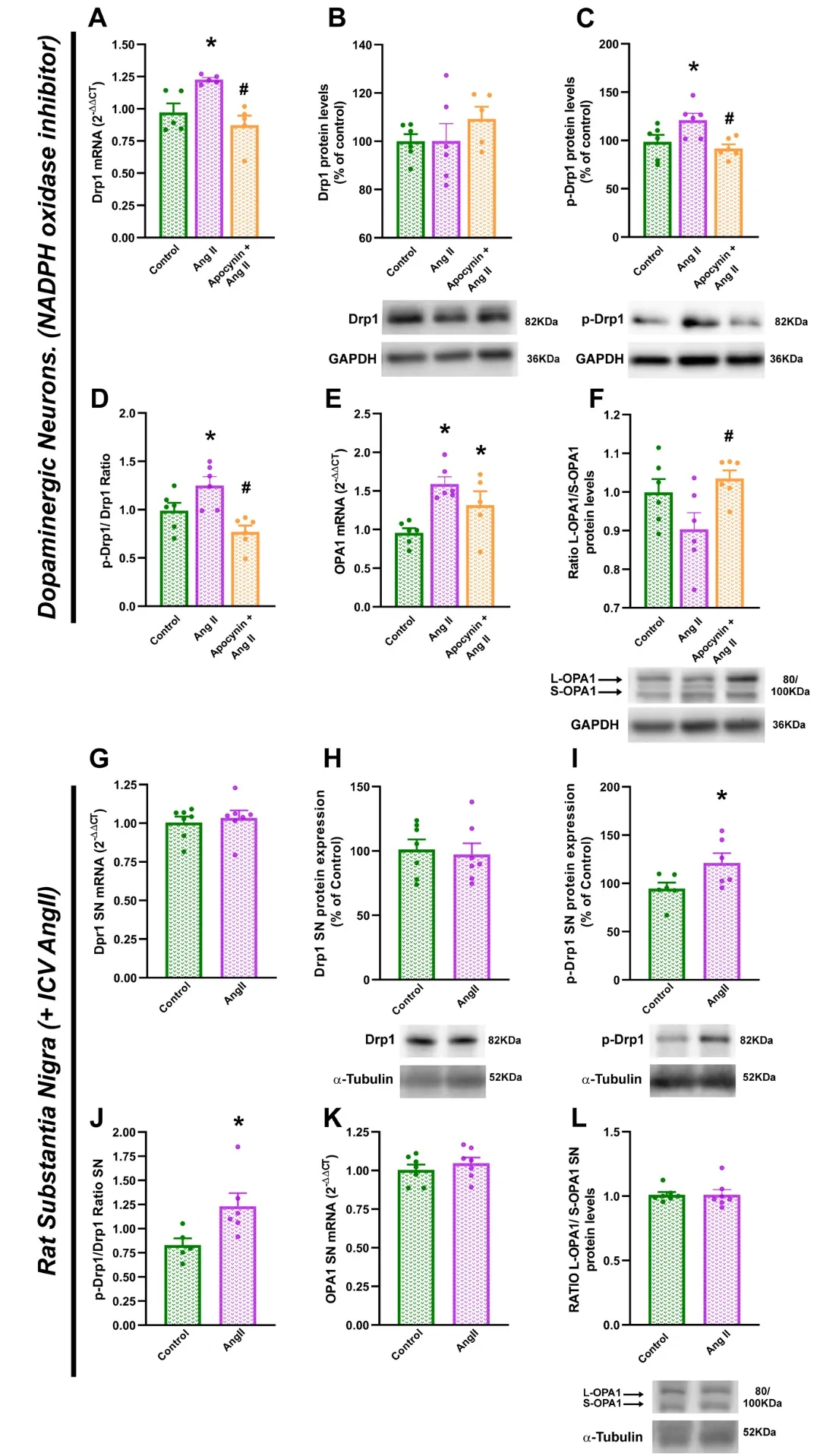
**NADPH oxidase complex mediates the effects Ang II on mitochondrial dynamics**. *In vivo* confirmation of Ang II effects. (A, C) Drp1 mRNA (N=5) and phosphorylated form of Drp1 at Serine 616 (p-Drp1) (N=6) were significantly increased in Ang II treated dopaminergic neurons but inhibited by pre-treatment with NADPH-oxidase complex inhibitor apocynin (B, D) Protein Drp1 levels were not significantly affected by Ang II or apocynin treatments, leading to an apocynin-dependent decrease in the p-Drp1/Drp1 ratio (N=6) (E) Fusion protein OPA1 mRNA was increased with Ang II treatment and not significantly inhibited by apocynin (N=5-6). (**F**) However, apocynin pre-treatment produced a significant increase in the ratio between the two forms of OPA1 (L-OPA1/S-OPA1) protein compared with Ang II alone (N=6). (**G-H**) Drp1 mRNA and total protein expression were not changed in tissue homogenate from SN of rats intraventricularly injected with Ang II relative to control rats (N=7). (**I-J**) However, an increase in Ser616 Drp1 phosphorylated form and, consequently, in p-Drp1/Drp1 ratio was observed. (**K-L**) In SN lysates, mRNA and protein expression of the two forms of fusion mediator OPA1 (L-OPA1/S-OPA1) were not modified by Ang II (N=7). Data are representative of three independent experiments and presented as mean ± SEM. * *p <* 0.05. One Way ANOVA with Student-Newman-Keuls Method post hoc test: A (P =0.003), B (P=0.421), C (P=0.012), D (P=0.002), E (P=0.004), F (P=0.040); Student’s t-test: G (P=0.631), H (P=0.744), I (P=0.048), J (P=0.034), K (P=0.405); Mann-Whitney Rank Sum Test: l (P=0.456). Abbreviations: Ang II, Angiotensin II; Drp1, dynamin-related protein 1; L-OPA1, long optic atrophy 1, S-OPA1, short optic atrophy 1; GAPDH, Glyceraldehyde-3-phosphate dehydrogenase; SN, substantia nigra, ICV, intraventricular injection.

Next, we used a potent inhibitor of the NADPH-oxidase complex, apocynin, to find out its role in the Ang II-dependent effect in mitochondrial dynamic processes. In Ang II-treated microglial cells, inhibition of NADPH-oxidase complex produced a decrease in Drp1 mRNA (Fig. 2A) and Ser616 Drp1phosphorylated protein, p-Drp1, and consequently in p-Drp1/Drp1ratio (Fig. 2C, D), implicating a direct role of the NADPH-oxidase complex in Ang II-induced fission related effects. Moreover, gene expression of fusion protein OPA1 was not affected by apocynin treatment compared with Ang II treatment alone (Fig. 2E), but apocynin changed the L-OPA1/S-OPA1 protein expression ratio, due to an increase in L-OPA1, which indicates that the inhibition of the NADPH-oxidase complex balances dynamic processes towards less fragmented mitochondria by both fission and fusion effectors (Fig. 2F).

### Mitochondrial fission is promoted by Ang II in the rat substantia nigra

Then, we studied the effect of Ang II in an *in vivo* model. We analysed the effect of intraventricular (icv) injection of Ang II on the expression of mitochondrial dynamic main proteins in the substantia nigra (SN). Significant changes in Drp1 mRNA or total protein expression were not

### Ang 1-7 inhibits the increase in mitochondrial fission in pro-inflammatory-activated microglial cells

**Figure 3.**
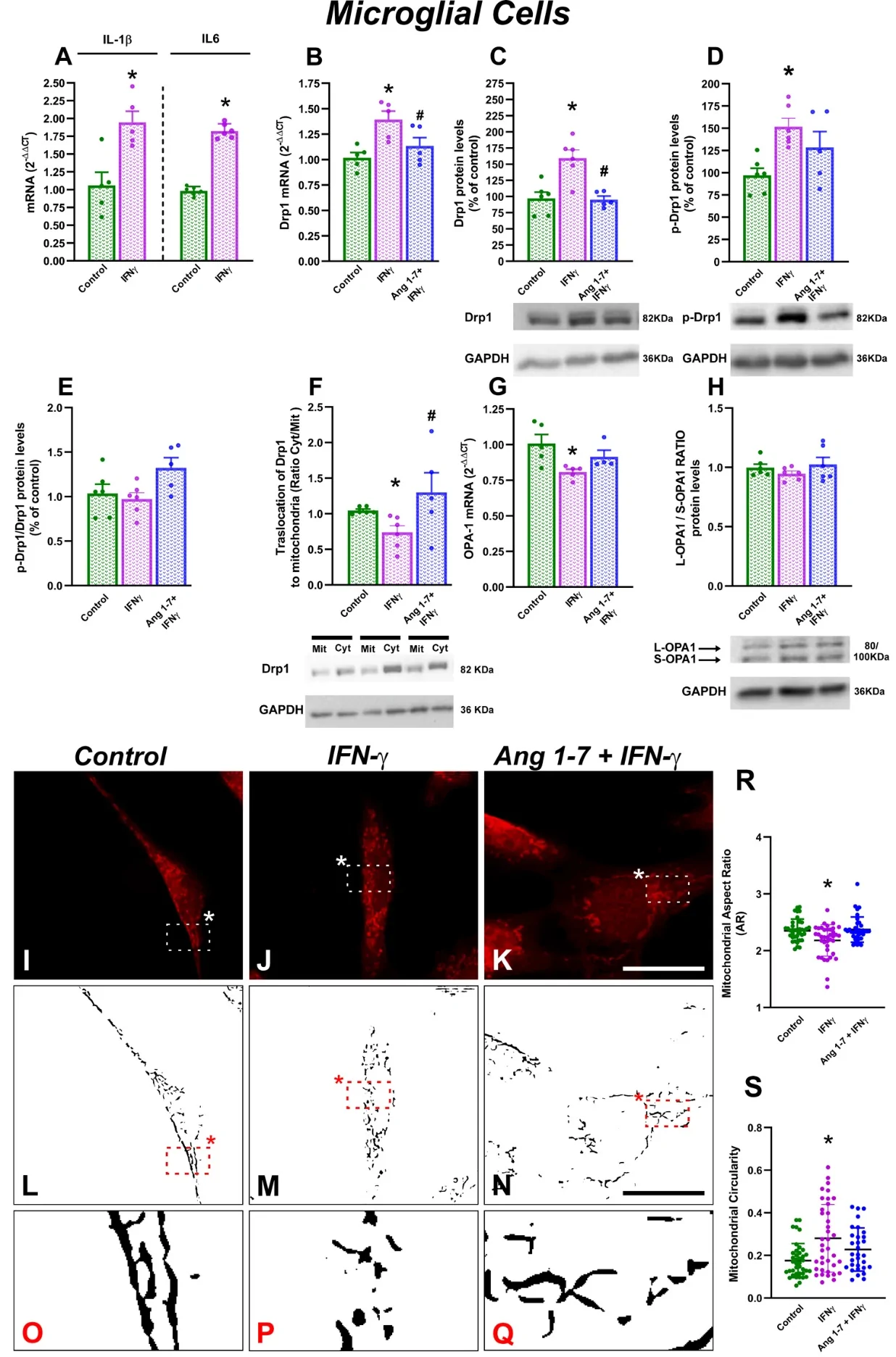
**Effect of Ang 1-7 on mitochondrial dynamics of proinflammatory microglia**. (**A**) Microglial cell treated with IFNγ produced a pro-inflammatory response shown by the increase in IL-1β and IL-6 mRNA relative to non-treated cells (N=5). (**B-E**) Drp1 mRNA (N=5), protein, and activated phosphorylated form of Drp1 at Serine 616 (p-Drp1) were significantly increased in IFNγ -treated cells, but not in the presence of Ang 1-7, leading to unaltered p-Drp1/Drp1 ratio. (**F**) IFNγ increased the translocation of Drp1 from the cytosol to mitochondria, as shown by the decrease in the ratio of cytosolic/mitochondrial protein levels, which was inhibited by Ang 1-7 (N=6). (**G-H**) OPA1 mRNA (N=4-5) was decreased by IFNγ treatment, which was inhibited by Ang 1-7, while L-OPA1/S-OPA1 ratio was not changed (N=6). (**I-Q**) Mitochondria were stained with MTDR (I-K), and images were processed and analysed for area, perimeter, major, and minor axis using Fiji/ImageJ software to calculate the mitochondrial shape descriptors aspect ratio and circularity (O-Q amplified boxed area). (**R-S**) IFNγ treatment produced a significant change in mitochondrial morphology towards higher fragmentation as shown by a decrease in aspect ratio [AR], and an increase in circularity [C]. Ang 1-7 pre-treatment induced more elongated mitochondrial morphology compared to IFNγ treatment alone. Ten pictures were analyzed for each treatment (N=31-40 cells per group). Data are representative of three independent experiments and presented as mean ± SEM *p < 0.05. Student’s t-test: A (P=0.006/P<0.001); One-way ANOVA followed by Student-Newman-Keuls method for multiple comparisons: B (P=0.010), C (P<0.001), E (P=0.112), G (P=0.031), H (P=0.400); Kruskal-Wallis one-way analysis of variance on ranks test followed by Dunn´s method as post hoc test: D (P=0.014), F (P=0.018), R (P=0.013), S (P=0.009). Scale bars: 25 μm (I-Q). Abbreviations: IFNγ, Interferon γ; Ang 1-7, Angiotensin 1-7; Drp1, dynamin-related protein 1; L-OPA1, long optic atrophy 1; S-OPA1, short optic atrophy 1; GAPDH, Glyceraldehyde-3-phosphate dehydrogenase; Mit, Mitochondrial fraction; Cyt, Cytosolic fraction.

We investigated whether the microglial pro-inflammatory response alters mitochondrial dynamics and the role of RAS in this process. First, we confirmed the pro-inflammatory activation of human microglial cell line HMC3 treated with Interferon-γ (IFNγ) for 24 h. IFNγ-activated microglial cells showed an increase in mRNA proinflammatory cytokines IL-1β and IL-6 (Fig. 3A). The mitochondrial dynamics were altered by IFNγ treatment, as revealed by an increase in both mRNA and protein expression of the mitochondrial fission protein Drp1 (Fig. 3B, C). The activated phosphorylated form of Drp1, at Ser616, was also increased, which led to an unaltered p-Drp1/Drp1 ratio (Fig. 3D, E). The imbalance towards excessive mitochondrial fission related to microglial pro-inflammatory activation was also supported by the significant decrease of cytosol/mitochondria Drp1 ratio, related to an increase in translocation of Drp1from cytosol to mitochondria (Fig. 3F). IFNγ treatment also induced a decrease in mitochondrial fusion OPA1 mRNA, although not at protein level (Fig. 3G, H). Interestingly, Ang 1-7 pre-treatment inhibited all the Drp1 changes induced by IFNγ treatment in microglial cells regarding mRNA, protein, phosphorylation, and increased translocation of Drp1, which reveals the role of the microglial RAS in mitochondrial dynamic changes related to neuroinflammation (Fig. 3A-F).

To confirm that the results obtained at the protein level were related to changes in mitochondrial morphology, we labeled microglial mitochondria with the specific probe MTDR and quantified the mitochondrial aspect ratio (AR) and circularity using confocal images and the Fiji Image J software (Fig. 3I-S). IFNγ-activated microglial cells showed more fragmented mitochondria (Fig. 3J, M, P) compared with non-treated microglia (Fig. 3I, L, O), as shown by a lower AR factor and higher circularity factor (Fig. 3R, S). Pre-treatment of the cells with Ang 1-7 before IFNγ-microglial activation inhibited the morphological changes supporting the antagonistic role of Ang 1-7 in this process (Fig. 3K, N, Q).

detected after analysis of tissue homogenate from SN of rats intraventricularly injected with Ang II (Fig. 2G, H). However, we observed an increase in Ser616 Drp1 phosphorylated form and, consequently, in p-Drp1/Drp1 ratio (Fig. 2I, J). In the SN, neither mRNA nor protein expression of the fusion mediator OPA1 (L-OPA1/S-OPA1 ratio) were affected by icv Ang II, supporting that Ang II alters mitochondrial dynamics mainly by acting on Drp1main fission protein (Fig. 2K, L).

### Ang 1-7 protects mitochondria against metabolic changes induced by pro-inflammatory microglial activation

Microglial and other immune cells change their metabolic pathways when they become activated in response to several immune insults. Main changes are produced due to the higher demand for ATP due to immune activation so that activated microglia increase glycolysis and decrease OXPHOS respiration [49].

**Figure 4.**
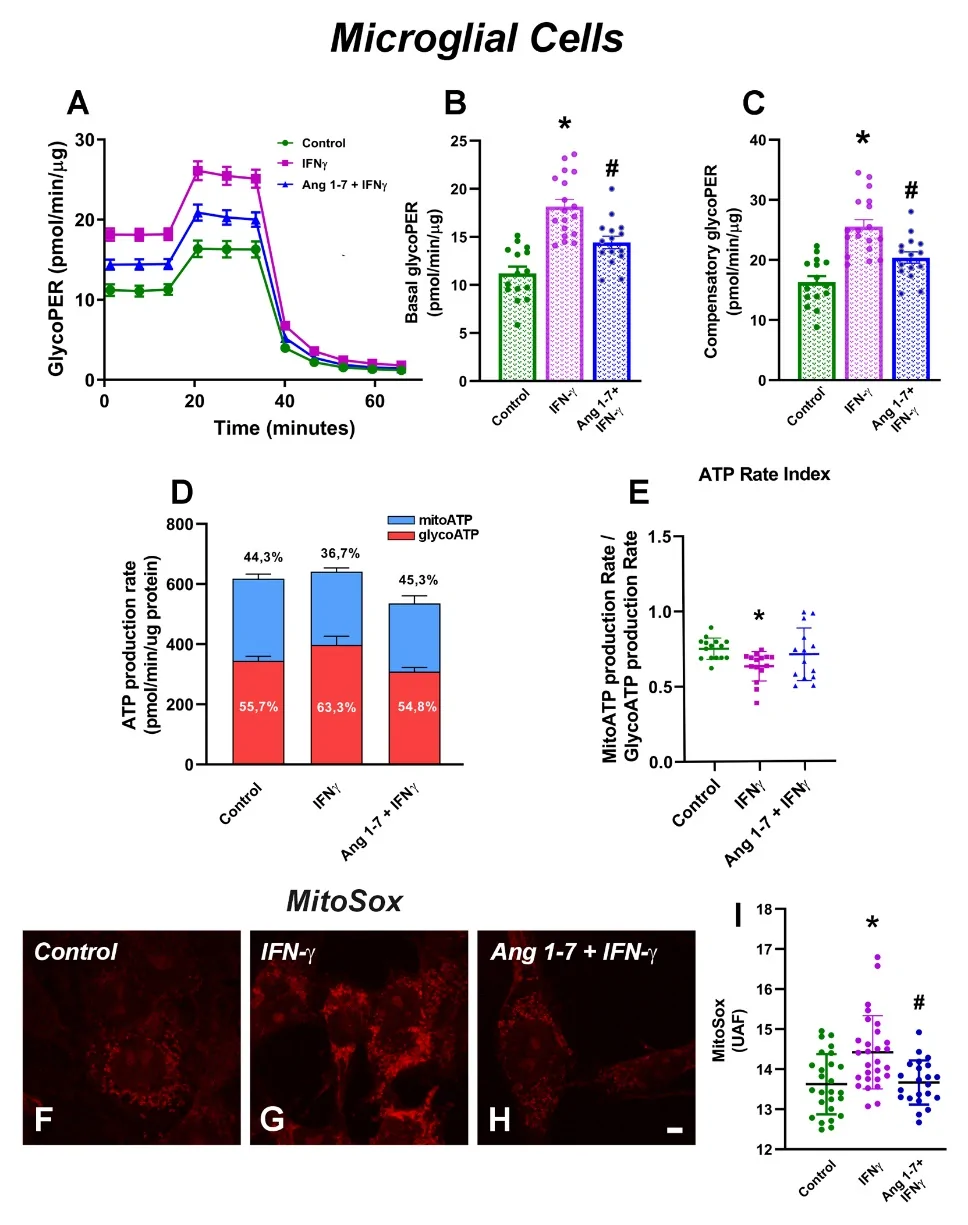
**Effect of Ang 1-7 on mitochondrial function in pro-inflammatory microglia**. (**A-C**) IFNγ activated microglia showed a significant increase in both basal and compensatory glycolysis. Administration of Ang 1-7 inhibited the excessive shift toward glycolytic metabolism (N=14-18). (**D-E**) No significant changes in total ATP rate with or without Ang 1-7 pre-treatment; however, we observed an increase in ATP production from glycolysis (GlycoATP) and a decrease in ATP production from mitochondrial respiration (MitoATP), which was inhibited by pretreatment with Ang 1-7, as shown in the ATP Rate Index (MitoATP/GlycoATP) (N=14-15). (**F-I**) In addition, IFNγ-treated microglial cells showed increased mitochondrial ROS production (MitoSox) relative to control cells, which was prevented by Ang1-7 pre-treatment (H, I) Ten pictures were analyzed for each treatment (N=22-28 cells per group). Data are representative of three independent experiments and presented as mean ±SEM. *p < 0.05. One-way ANOVA followed by Student-Newman-Keuls method for multiple comparisons: B (P=0.018), C (P<0.001); Kruskal-Wallis one-way analysis of variance on ranks test followed by Dunn´s method as post hoc test: E (P=0.018), I (P=0.001). Scale bars: 25 μm (F-H). Abbreviations: IFNγ, Interferon γ; Ang 1-7, Angiotensin 1-7; GlycoPER, Glycolytic proton efflux rate; MitoATP, ATP from mitochondria; GlycoATP, ATP from glycolysis.

We measured the metabolic state of HMC3 microglial cells by calculating the Glycolytic Proton Efflux Rate (glycoPER; rate of protons liberated to the medium during glycolysis) in our experimental conditions using a Seahorse metabolic analyser. We observed that IFNγ- activated microglia showed a significant increase in both basal (rate of glycolysis under basal conditions) and compensatory (capability to compensate energy demands of the cell with mitochondrial inhibition) glycolysis (Fig. 4A-C), confirming the shift to glycolysis-based metabolism in response to inflammatory stimulus. Pre-treatment of the activated microglial cells with Ang 1-7 inhibited the excessive shift toward glycolytic metabolism (Fig. 4A-C). We also measured the ATP production rate in living cells using a Seahorse metabolic analyser, which simultaneously measures basal ATP production rates from mitochondrial respiration and glycolysis. We observed no significant changes in total ATP rate but an increase in ATP production from glycolysis (GlycoATP) and a decrease in ATP production from mitochondrial respiration (MitoATP) in IFNγ treated cells, which was inhibited in IFNγ-stimulated cells previously treated with Ang 1-7, as represented in the ATP Rate Index (MitoATP/GlycoATP) (Fig. 4D, E). The results suggest that immuno-activated microglial cells compensate for the decrease in mitoATP production with an increase in glycolytic activity, with no changes in total ATP production. Interestingly, pre-treatment with Ang1-7 prevents mitochondrial ROS changes related to the activation of microglia (Fig. 4F-I). In IFNγ-treated microglial cells, we observed an increase in mitochondrial ROS production (Fig. 4G) relative to non-treated cells (Fig. 4F), as measured by the specific probe MitoSox. These changes were abolished when we pre-treated the cells with Ang 1-7 for 30 min before IFNγ treatment (Fig. 4H, I).

### Deletion of angiotensin receptors affects mitochondrial dynamic changes related to neuroinflammation

**Figure 5.**
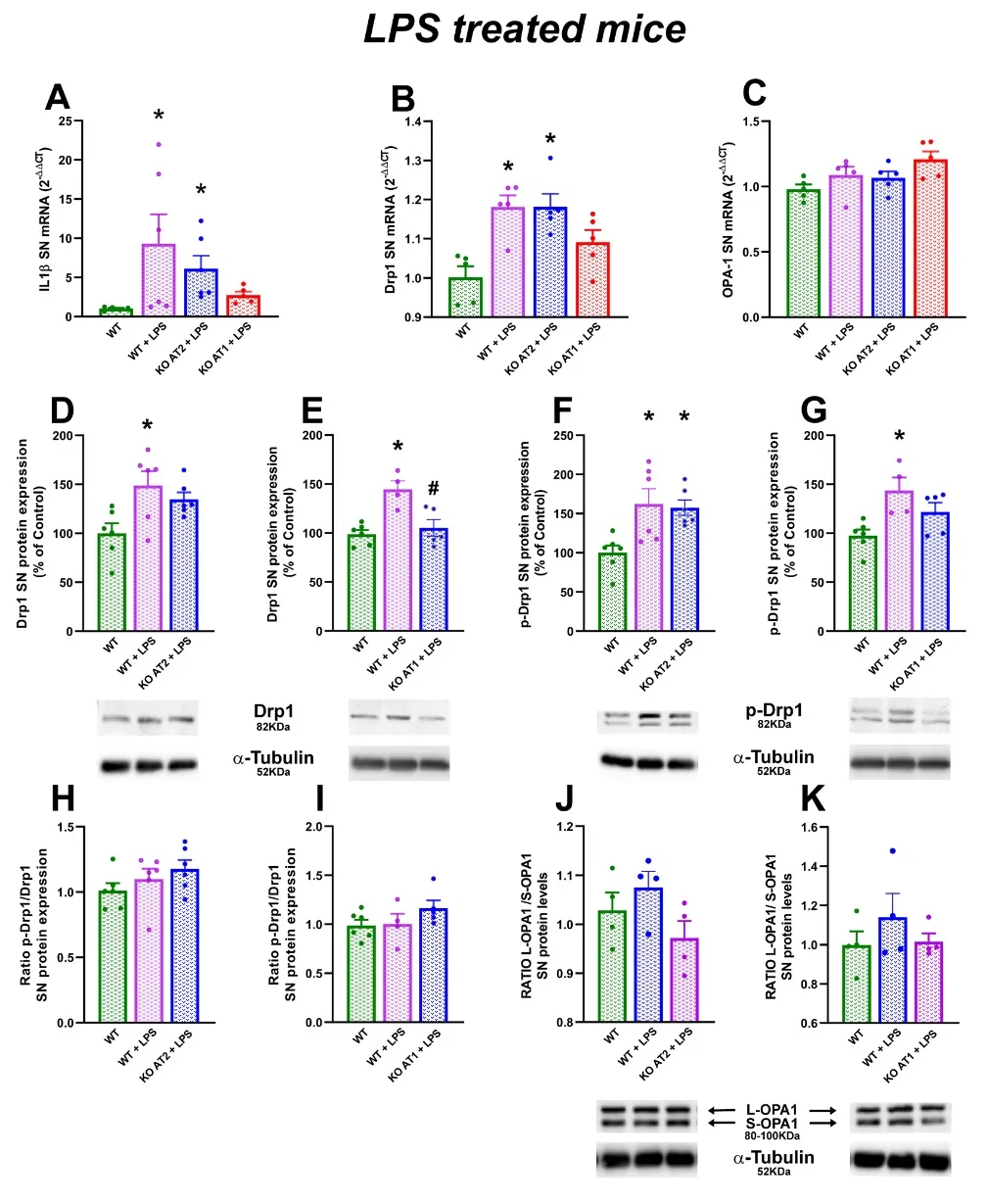
**Mitochondrial dynamics in Substantia Nigra from LPS-treated AT1 and AT2 receptor-deficient mice**. (**A**) Levels of pro-inflammatory cytokine IL1-β mRNA (B, D-E) Drp1 mRNA and protein, and (F-G) activated phosphorylated form of Drp1 at Serine 616 (p-Drp1) were significantly increased in WT and AT2KO LPS- injected mice but not in AT1KO LPS- injected mice compared with WT mice (H-I) Consistent with this, p-Drp1/Drp1 ratio was not significantly changed. (C, J-K) OPA1 mRNA and L-OPA1/S-OPA1 protein ratio were not significantly changed (N=4-6/group, relative expression to WT, as a control group). Data are representative of three independent experiments and presented as mean ± SEM *P < 0.05; One-way ANOVA followed by Student-Newman-Keuls method for multiple comparisons: B (P=0.002), C (P=00.53), D (P=0.020), E (P=0.002), G (P=0.015), H (P=0.272), I (P=0.232), J (P=0.163), K (P=0.456); Kruskal-Wallis One Way Analysis of Variance on Ranks: A (P=0.010), F (P=0.008). Abbreviations: drp1, Dynamin-related protein 1; L-OPA1, long optic atrophy 1; S-OPA1, short optic atrophy 1; LPS, lipopolysaccharide; WT, wild-type; AT1KO, Angiotensin II receptor type 1 deficient mice; AT2KO, Angiotensin II receptor type 2 deficient mice; SN, Substantia nigra.

We next investigated the effect of deletion of Ang II receptors of the pro-oxidative and anti-oxidative RAS arms on mitochondrial dynamics using the LPS inflammatory mice model. In the SN of wild-type (WT) and AT2 deficient mice (AT2KO), LPS treatment produced an increase in mRNA expression of the proinflammatory cytokine IL1β; however, no significant changes were observed AT1 deficient mice (AT1KO) (Fig. 5A).

Regarding mitochondrial dynamic processes, major changes were observed in the main fission protein Drp1 in the SN. We observed that Drp1 mRNA, total and phosphorylated Drp1 protein were significantly increased by LPS treatment in WT and AT2KO mice relative to untreated WT mice; however, these LPS-induced increases were not observed in AT1KO mice, (Fig. 5B, D-I). Consistent with the *in vitro* data, OPA1 mRNA and protein levels were not altered in any of the mouse models (Fig. 5C, J-K), which further confirms that the main effects of Ang II on inflammation-related mitochondrial dynamic changes are induced *via* AT1 receptors and Drp1 fission protein.

### Ang 1-7 treatment modulates phosphorylation and distribution of the transcription factor STAT3 in pro-inflammatory activated microglia

**Figure 6.**
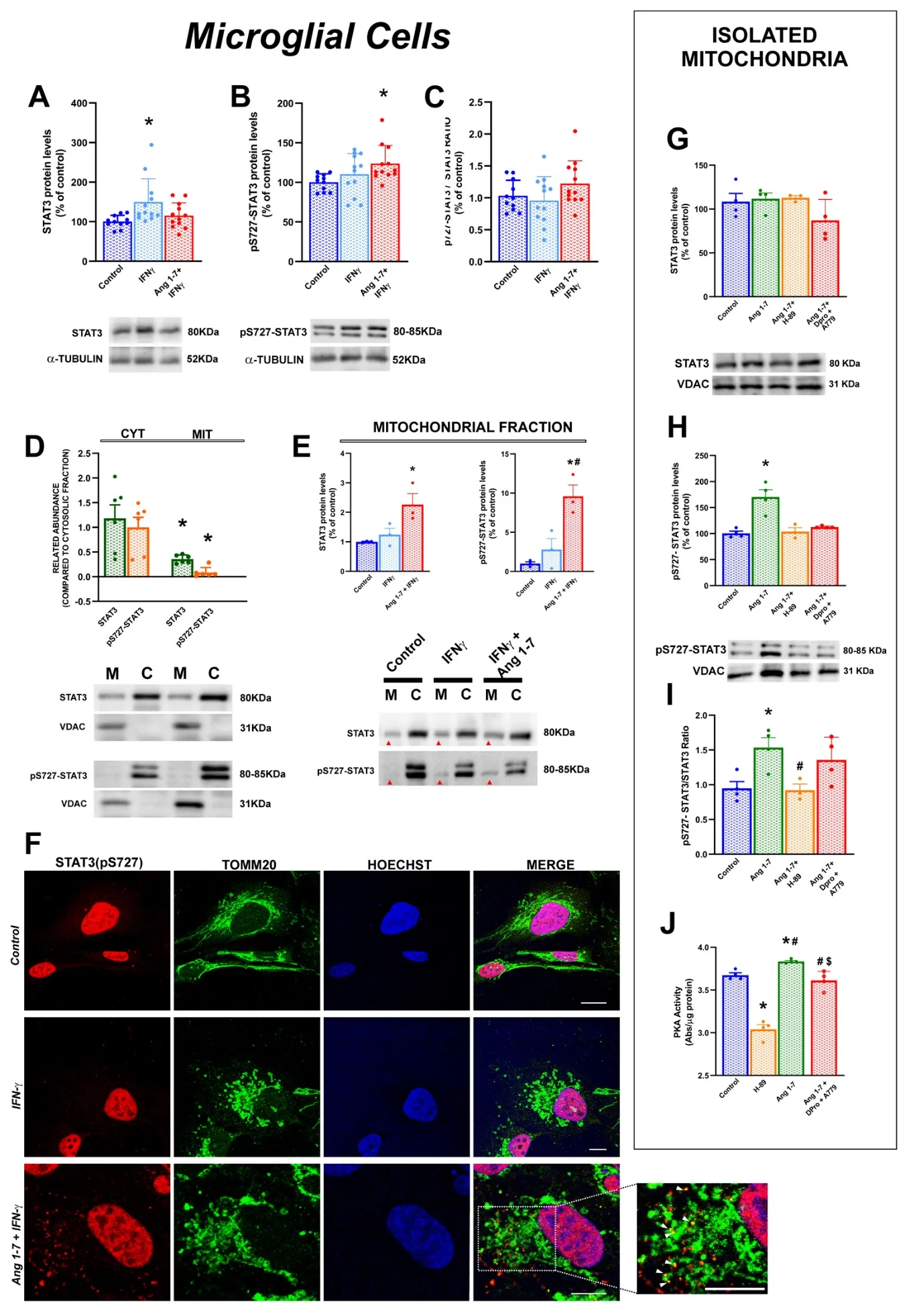
**Ang 1-7 increased the non-canonical phosphorylated p-S727 STAT3 form at cellular and mitochondrial levels**. (**A**) In microglial cells treated with IFNγ, STAT3 protein levels were significantly increased, and were inhibited by Ang 1-7 (B) p-S727 STAT3 was significantly upregulated by Ang 1-7. (**C**) p-S727 STAT3/STAT3 ratio was slightly but not significantly increased in the Ang 1-7 + IFNγ group (N=11-12). (**D**) Mitochondrial levels of unphosphorylated STAT3 were low relative to cytosolic levels, and pS727-STAT3 showed almost no mitochondrial expression compared with the cytosolic expression (N=6). (**E**) In the mitochondrial fraction, Ang 1-7 significantly increased both STAT3 and pS727-STAT3 levels (around a 10-time fold increase) (N=3). (**F**) Double immunofluorescence showed the nuclear location of pS727-STAT3 in control and INF-γ-treated cells. Ang 1-7 treated cells showed a clear cytosolic presence of pS727-STAT3, partially colocalizing with mitochondrial TOMM20 (magnified in the boxed area). (**G-I**) Isolated mitochondria treated with Ang 1-7 showed no changes in the unphosphorylated STAT3 form but a significant increase in pS727-STAT3 protein expression, which increased the pS727-STAT3/STAT3 ratio. Mitochondrial Ang 1-7 effects were not produced in the presence of MAS and MrgE receptor antagonists (A779 and D-Pro) or the PKA inhibitor H-89. (**J**) In isolated mitochondria, Ang 1-7 increased PKA activity, which was inhibited by MAS and MrgE antagonists, and by the specific PKA inhibitor H-89 (N=3-4). Data are representative of three independent experiments and presented as mean ± SEM. *P<0.05; One Way ANOVA with Student-Newman-Keuls Method post hoc test: B (P=0.039), C (P=0.147), E (P=0.026/P=0.005), G (P=0.188), I (P=0.017), J (P<0.001); Kruskal-Wallis One Way Analysis of Variance on Ranks with Dunn's Method post hoc test: A (P=0.011), H (P=0.016). Mann-Whitney Rank Sum Test: D (P=0.015/P<0.001). Scale bars: 10 μm (F). Abbreviations: Ang 1-7, Angiotensin 1-7; STAT3, signal transducer and activator of transcription; pS727-STAT3, signal transducer and activator of transcription phosphorylated in Serine 727; VDAC, voltage-dependent anion channel; Dpro, D-Proline; A779, D-Ala7-Ang-(1-7); H-89 dihydrochloride.

To identify potential mediators of the effects of Ang 1-7 on mitochondrial dynamics, we studied the possible link between Ang 1-7 and the transcription factor STAT3, in our model of inflammatory response using microglial cells, particularly, the non-canonical form of STAT3, phosphorylated at Serine 727 (pS727-STAT3), due to its known role as a mitochondrial function modulator [50]. First, we studied the effects of Ang 1-7 treatment on STAT3 expression in inflammatory microglial cells. We observed a significant increase in unphosphorylated STAT3 expression with IFNγ treatment, but not in the presence of Ang 1-7 pre-treatment (Fig. 6A). Interestingly, the non-canonical form of STAT3, pS727-STAT3, showed higher expression with Ang 1-7 treatment (Fig. 6B, C), which reveals a direct role of Ang 1-7 in this type of phosphorylation of STAT3.

The non-canonical pathway of STAT3 might translocate to mitochondria and be involved in several mitochondrial functions [50], so we checked the expression of the unphosphorylated STAT3 form and S727 phosphorylated form of STAT3 (pS727-STAT3), under basal conditions, on the mitochondrial fraction of microglial cells. In basal conditions, we observed a low but constitutive expression of STAT3 in mitochondria; however, pS727-STAT3 showed almost no mitochondrial expression compared with the cytosolic expression (Fig. 6D, E). Then, we studied the effect of the Ang 1-7 treatment on the mitochondrial levels of STAT3 and pS727-STAT3. We observed a marked increase in both, STAT3 and pS727-STAT3 in the mitochondrial fraction of IFNγ -activated microglia treated with Ang 1-7, the latter especially marked (near 10-time fold increase) (Fig. 6E), suggesting a link between Ang 1-7 and the STAT3 role at the mitochondrial level. To confirm these data and localize the proteins at the subcellular level, we performed immunofluorescence assays with nuclear and mitochondrial labeling in control and treated microglial cells (Fig. 6F). We observed a clear, almost exclusive, nuclear location of pS727-STAT3 in control and IFNγ-treated cells. However, a clear change in distribution to a more cytoplasmic presence was observed when the cells were pre-treated with Ang 1-7 before IFNγ treatment, showing at least partial colocalization of pS727-STAT3 with the mitochondrial labeling (Fig. 6F). The presence of pS727 STAT3 at the cytoplasmic level outside the mitochondrial pattern observed after Ang 1-7 treatment might be related with the observed mitochondrial dynamic changes. Physical association of pS727 STAT3 with microtubules has been suggested to be involved in the maintenance of mitochondrial dynamics [51, 52]. Regarding unphosphorylated STAT3, changes were less evident. Both control and IFNγ-treated cells, with and without Ang 1-7 pre-treatment, showed a cytoplasmic distribution of the protein.

### In isolated mitochondria, Ang 1-7 increases intramitochondrial phosphorylation of STAT3 at Ser727 via mitochondrial PKA

The fact that the unphosphorylated STAT3 form was increased at the mitochondrial level after Ang 1-7 pre-treatment led us to investigate the possible intramitochondrial phosphorylation of this molecule, as it was shown that, inside the mitochondria, the main functional effects are induced by the Ser727 phosphorylated form [53]. In previous studies, we showed the effects of mitochondrial Ang 1-7 receptors, (i.e. activation of mitochondrial MasR and, particularly, MrgE receptors) on mitochondrial parameters such as mitochondrial NO/ROS production [21, 23, 38]. Therefore, we checked whether the activation of these receptors might be involved. To this end, we treated isolated mitochondria from microglial cells with Ang 1-7 to activate the mitochondrial receptors as in our previous studies. We observed a significant increase in pS727-STAT3 expression in isolated mitochondria treated with Ang 1-7, but not for the unphosphorylated STAT3 form, which increased the pS727-STAT3/STAT3 ratio. We did not observe Ang 1-7 effects when we blocked mitochondrial Ang 1-7 receptors with the MAS and MrgE receptors antagonists A779 and D-Pro, respectively (Fig. 6G, I). In addition, as shown for other mitochondrial G-coupled receptors [54], intramitochondrial PKA activity can be modified by intramitochondrial signaling. Activation of intramitochondrial PKA affects mitochondrial function by phosphorylating different targets such as OXPHOS proteins, apoptosis-related proteins, and dynamics [53, 55, 56]. To test whether PKA could be involved in the intramitochondrial phosphorylation of STAT3, we pre-treated isolated Ang 1-7-treated mitochondria with the specific inhibitor of PKA H-89, which corroborated the role of this enzyme in Ang 1-7 effect (Fig. 6G, I). Finally, we measured PKA activity in isolated mitochondria treated or not treated with Ang 1-7, with or without the Ang1-7 receptor inhibitors A779 and D-Pro. In the present work, we observed an increase in mitochondrial PKA activity in isolated mitochondria from microglial cells treated with Ang 1-7 compared with non-treated isolated mitochondria. Moreover, the increase in PKA activity was not observed when we pre-treated isolated mitochondria with A779 and D-Pro to block mitochondrial Ang 1-7 receptors (MasR and MrgE). As an internal control, PKA activity was confirmed with the use of the specific PKA inhibitor, H-89 (Fig. 6J).

### IL-10 mediates Angiotensin 1-7 effects on mitochondrial dynamics and STAT3 phosphorylation at Ser727

**Figure 7.**
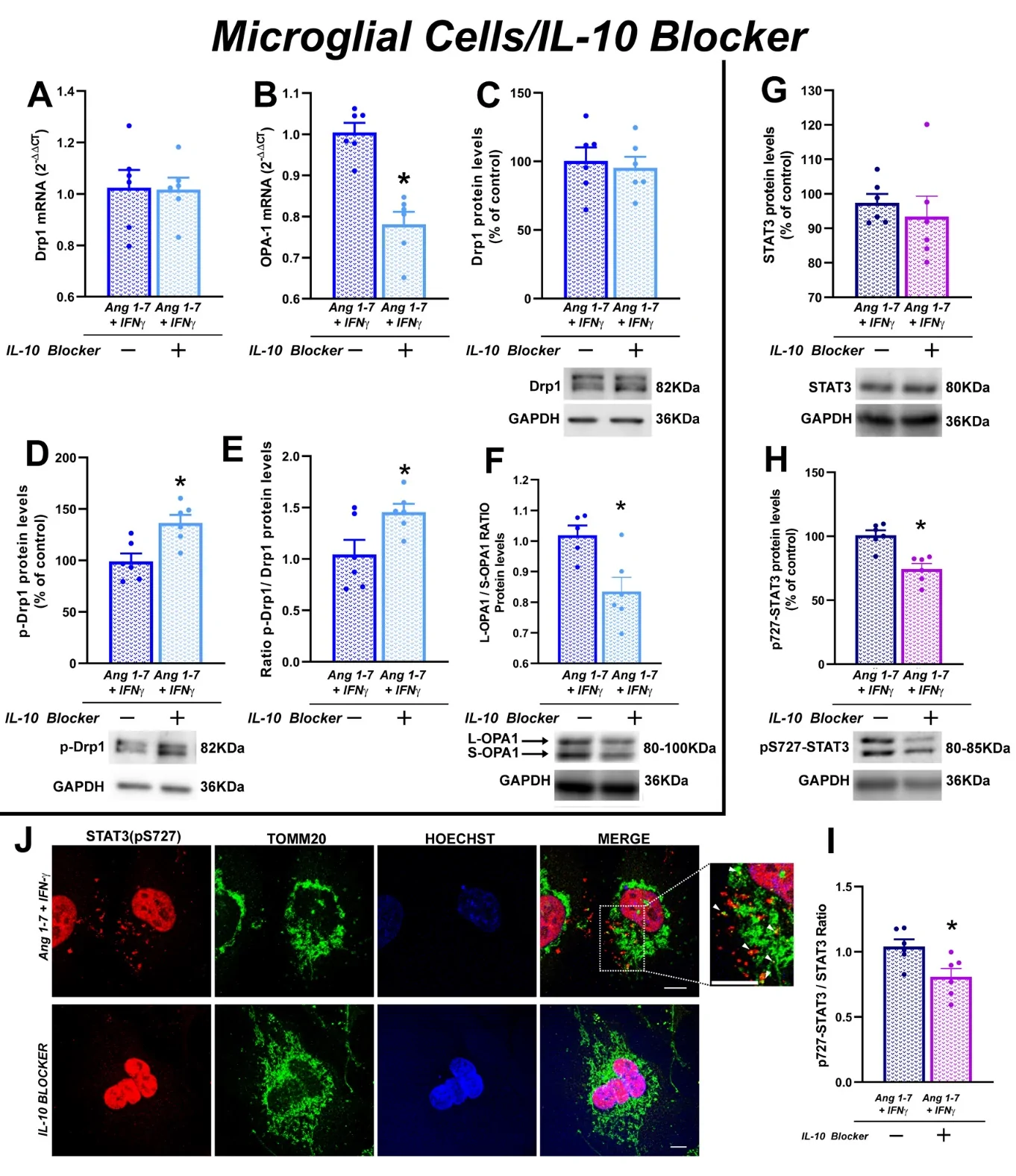
**Effect of IL-10 blockage on mitochondrial dynamics and STAT3 in pro-inflammatory microglia**. (A, C) Functional blockage of IL-10 in Ang 1-7 + IFNγ microglial cells did not produce significant changes in Drp1 mRNA or protein. (D, E) However, it increased the activated phosphorylated form of Drp1 at Serine 616 (p-Drp1), leading to an increased p-Drp1/Drp1 ratio. (B, F) Both, OPA1 mRNA and L-OPA1/S-OPA1 protein ratio were significantly decreased after the blockage of IL-10 (G-H) Blockage of IL-10 did not change unphosphorylated STAT3 protein levels but decreased significantly pS727-STAT3 and the ratio between both STAT3 forms. (N=5-6; relative expression to Ang 1-7 + INFγ, as a control group). (**J**) IL-10 blockage induced a clear change in pS727-STAT3 location as the cytosolic and mitochondrial presence of pS727-STAT3 (top row) was absent in cells pretreated with the IL-10 blocking antibody (bottom row); the boxed area is magnified in a single confocal microscopy plane image. Data are representative of three independent experiments and presented as mean ± SEM *P < 0.05. Student’s t-test: A (P=0.932), B (P<0.001), C (P=0.699), D (P=0.007), E (P=0.029), F (P=0.012), G (P=0.552), I (P=0.019); Mann-Whitney Rank Sum Test: H (P=0.002). Scale bars: 10 μm (F). Abbreviations: IL-10, Interleukin 10; Drp1, dynamin-related protein 1; L-OPA1, long optic atrophy 1; S-OPA1, short optic atrophy 1; STAT3, signal transducer and activator of transcription; pS727-STAT3, signal transducer and activator of transcription phosphorylated in Serine 727; TOMM20, translocase of outer mitochondrial membrane 20.

A link between some components of the RAS protective arm (AT2 receptors or Ang 1-7) and the anti-inflammatory cytokine IL-10 was observed in several tissues and cells, including microglia [40, 57-59]. We therefore studied whether IL-10 may mediate the effects of Ang 1-7 on mitochondrial dynamic changes induced by pro-inflammatory microglial activation. In IFNγ-activated microglia treated with Ang 1-7, we blocked IL-10 with an excess of anti-IL10 antibodies [43]. In the absence of IL-10 signaling, total Drp1 mRNA or protein expression were not affected (Fig. 7A, C). However, we observed a significant increase in the expression of p-Drp1 protein, and consequently the ratio p-Drp1/Drp1 (Fig. 7D, E). Therefore, the effect of Ang 1-7 on decreasing the phosphorylated form of Drp1 was suppressed when IL-10 was not functional. Interestingly, in IFNγ-activated microglia treated with Ang 1-7, the blockage of IL-10 produced a significant decrease in both mRNA and ratio of two forms of the main fusion protein OPA1, which implies that IL-10 also regulates mitochondrial dynamics *via* OPA1 (Fig. 7B, F).

Next, we investigated the role of IL-10 inhibition on the effects of Ang 1-7 on the expression of the transcription factor STAT3. The blockage of IL-10 produced a significant decrease in the phosphorylated form pS727-STAT3, without changing the unphosphorylated STAT3, leading to a significantly lower pS727-STAT3/STAT3 ratio (Fig. 7G-I). These results confirm that the effect of Ang 1-7 on STAT3 is also mediated by IL-10 signalling. In addition, to find out if IL-10 is involved in the Ang 1-7-induced STAT3 cytoplasmic/mitochondrial translocation, we performed immunofluorescence studies of the STAT3 transcription factor with IL-10 blockage. In Ang 1-7-treated proinflammatory microglial cells, we observed cytoplasmic pS727-STAT3 only when IL-10 signalling was functional (Fig. 7J, 6F).

## DISCUSSION

**Figure 8.**
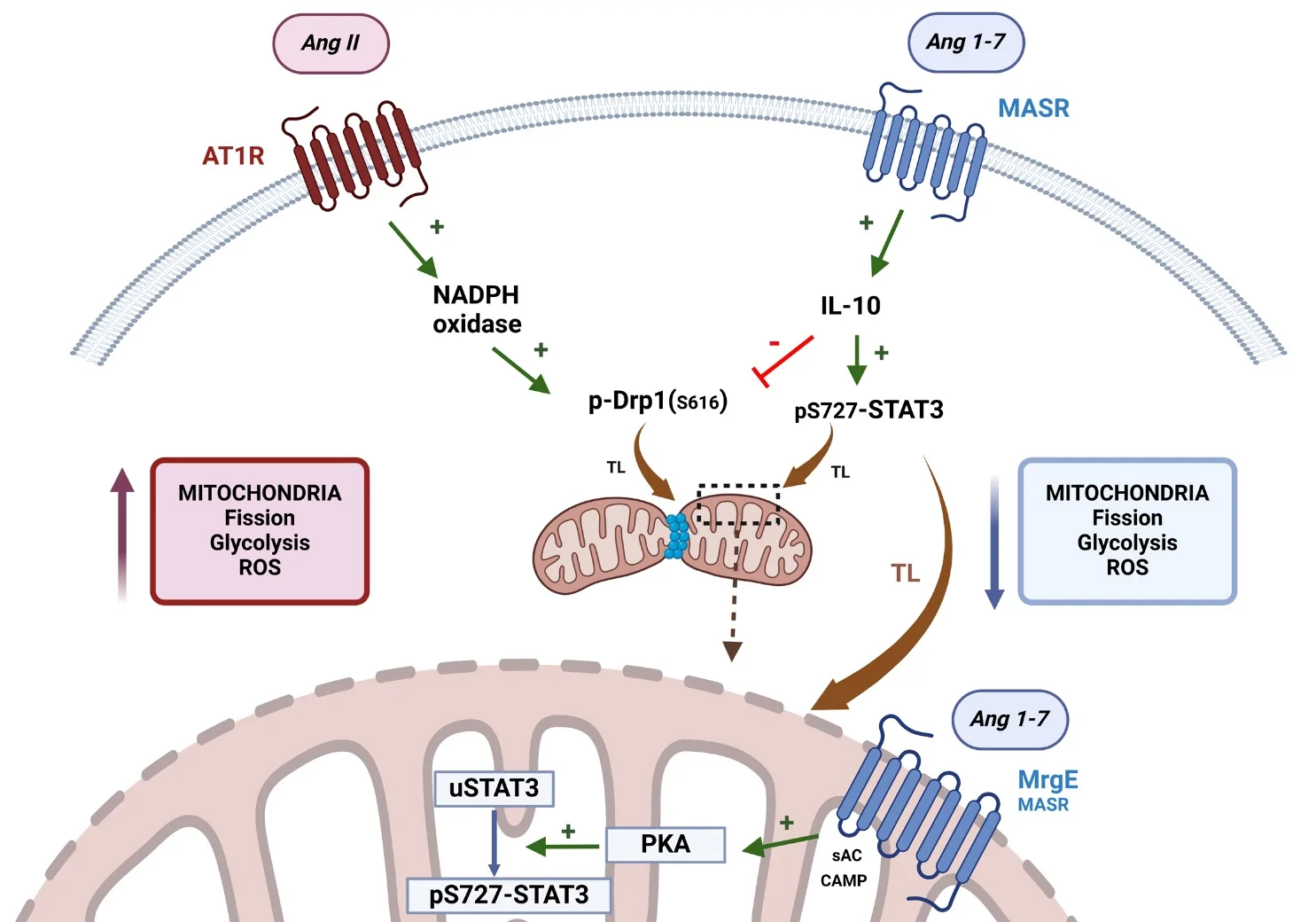
**Schematic model of the role of RAS in modulating mitochondrial dynamics and function in brain cells**. RAS regulates the mitochondrial function in neurons and microglial cells through mitochondrial dynamic regulation and at the respiratory level. Ang II/AT1 activation produces a dysregulation of mitochondrial dynamic processes towards an increase in mitochondrial fission, via Drp1 phosphorylation at Ser616 and translocation to mitochondria through NADPH oxidase complex. Ang 1-7 counteract the oxidation and inflammation-induced mitochondrial changes, decreasing mitochondrial fission, glycolysis, and ROS production through IL-10 and the non-canonical mitochondrial STAT3 pathway. Activation of mitochondrial Ang 1-7 receptors (i.e. MasR/MrgE) increases mitochondrial phosphorylation of STAT3 via increased mitochondrial PKA activity, which is responsible for Ang 1-7 effects at the mitochondrial level. Abbreviations: Ang II, Angiotensin II; Ang 1-7, Angiotensin 1-7; AT1R, angiotensin II type 1 receptor; MASR, Mas receptor; MrgE, Mas-related G-protein coupled receptor member E; Drp1, dynamin-related protein; IL-10, Interleukin 10; pS727-STAT3, signal transducer and activator of transcription phosphorylated in Serine 727; TL, translocation. The image was created using BioRender.com.

In the present study, we have shown the role of major components of the pro-oxidative/proinflammatory RAS (Ang II/AT1) and the anti-oxidative/anti-inflammatory RAS (Ang 1-7) in the mitochondrial dynamics in dopaminergic neurons and the inflammatory response of microglial cells. Furthermore, we showed that the effects are mainly via Drp1 modulation and that IL-10, the mitochondrial STAT3 pathway, and the mitochondrial PKA are involved in these effects (Fig. 8). The results show a new mechanism of action of the RAS on the mitochondrial function. Mitochondrial dynamics are essential for maintaining neuronal energy demands, and excessive mitochondrial fission was pointed as an early pathological event in toxin-induced cell death in PD models [60]. The imbalance in mitochondrial fission and fusion affects main mitochondrial functions, such as respiration, mitochondrial quality control, and apoptosis. It was demonstrated that Drp1-mediated mitochondrial fission facilitates cytochrome c release from mitochondria, playing a major role in eliminating damaged cells through apoptosis [61]. Drp1 is highly expressed in the brain, and post-translational modifications of Drp1 were shown to modulate neuronal cell death. Interestingly, adequate Drp1 modulation is considered essential for the mitochondrial function in neurons, and disequilibrium in mitochondrial dynamics, towards fission, was identified as an important mechanism responsible for the vulnerability of dopaminergic neurons in PD, and, consistent with this, inhibition of excessive mitochondrial fission, using a Drp1 blocker, led to neuroprotection [62].

In the paracrine or tissue brain RAS, the precursor protein angiotensinogen is mostly produced by astrocytes [63, 64], and the angiotensinogen-derived peptides of the pro- and anti-oxidative/inflammatory arms (Ang II and Ang 1-7, respectively) act on brain cells binding plasma membrane receptors such as the pro-oxidative AT1 and the anti-oxidative AT2 and Mas receptors [9, 18]. AT1 receptors are particularly abundant at the cell surface and promote the increase in intracellular ROS levels via activation of the plasma membrane NADPH-oxidase complex (i.e., Nox2, the second major intracellular source of superoxide after the mitochondria) [65]. Furthermore, previous studies in different cell types [66, 67], including dopaminergic neurons [68-70], have shown that ROS derived from the Nox2-released superoxide induce opening of mitochondrial ATP-sensitive potassium channels (mitoKATP), which increases the generation of mitochondrial ROS. The compensatory antioxidant receptors, AT2 and Mas, appear less relevant at the plasma membrane level [24, 38]. However, using isolated mitochondria, we have shown the presence of RAS receptors in the mitochondria, and that, opposite to that observed at the plasma membrane, the antioxidative RAS receptors (AT2 and Mas/Mas-related receptors) are much more abundant than AT1 receptors in the mitochondria, suggesting that they counteract at the mitochondrial level the pro-oxidative effects of the plasma membrane and mitochondrial AT1 receptors [22, 23, 38]. Surprisingly, we observed that the Mas-related receptor MrgE is the most abundant receptor at the mitochondrial level and that ACE2, which generates Ang 1-7 from Ang II, is also very abundant in mitochondrial preparations [21]. In the mitochondria, different angiotensin receptors modulate oxidative phosphorylation. Mitochondrial AT1 receptors, by activating mitochondrial Nox4, contribute to superoxide production. However, the much more abundant receptors of the antioxidative system induce, via nitric oxide, the downregulation of mitochondrial respiration and modulate oxidative phosphorylation [21, 23].

In the present study, we have shown that Ang II/AT1 activation produces a dysregulation of mitochondrial dynamic processes towards an increase in mitochondrial fission, via Drp1 phosphorylation at Ser616 and translocation to mitochondria, which is consistent with our previous data showing an Ang II-dependent increase in mitochondrial superoxide production [23]. Furthermore, consistent with the protective effects of Ang 1-7 observed in our previous functional studies [21, 38], we now showed that Ang 1-7 pre-treatment protects mitochondrial dopaminergic neurons against the Ang II-induced mitochondrial dynamic changes. Moreover, we identified the NADPH oxidase complex as an essential mediator of Ang II effect on mitochondrial fission.

It is known that the microglial neuroinflammatory response induces the release of reactive oxygen (ROS), nitrogen species (RNS), and pro-inflammatory cytokines, which play a major role in the dopaminergic neuron death and the progression of PD [35, 71]. Previous studies from our group and others have shown the implication of the brain RAS in the modulation of the microglial pro-inflammatory responses, through regulation of inflammation-related changes in microglial NADPH-oxidase, Rho-kinase and the inflammasome complex [34, 35, 72, 73]. Alternations between different microglial states are highly energy-consuming processes and entail metabolic and morphological changes, where the role of mitochondria is essential. Furthermore, mitochondrial dysfunction in microglia has been associated with enhanced pro-inflammatory states and higher release of neurotoxic pro-inflammatory cytokines [74]. In the present study, we revealed that the brain RAS modulates the microglial pro-inflammatory response through mitochondrial dynamic changes. After IFNγ-induced activation of human microglial cells, we observed increased mitochondrial fragmentation and mitochondrial superoxide production. Interestingly, pre-treatment of cells with Ang 1-7 inhibited the IFNγ-induced mitochondrial changes, thus demonstrating the role of this RAS component in regulation of mitochondrial alterations related to the microglial inflammatory response.

Furthermore, we investigated the effects of Ang 1-7 treatment on the metabolic shift of microglial cells in response to inflammatory stimuli. To supply the higher demand for ATP energy for the microglial inflammatory response, a change in mitochondrial metabolism from OXPHOS preference to glycolysis-based metabolism takes place [49]. We observed that Ang 1-7 also inhibits this metabolic switch in IFNγ-treated microglial cells, at basal and compensatory glycoPER.

The role of RAS in mitochondrial dynamic changes related to neuroinflammation was confirmed *in vivo* using the LPS-induced inflammation model in WT mice compared with mice deficient in receptors of the RAS pro-oxidative or anti-oxidative axis (AT1KO and AT2KO mice, respectively). We observed that LPS produced an increase in total and phosphorylated form of the main mitochondrial fission protein Drp1 in WT and AT2KO mice, which was not observed in LPS-treated AT1KO mice. AT1KO mice are characterized by a down-regulation of the pro-inflammatory RAS axis with an imbalance towards the anti-inflammatory axis [38, 40]. Our *in vivo* data further support the role of RAS in mitochondrial dynamic changes related to the inflammatory response since, in the absence of the main RAS pro-oxidative/pro-inflammatory receptor (AT1), mitochondria are protected from the increase in mitochondrial fragmentation that occurs in WT and AT2KO mice.

A link between the protective effects of anti-inflammatory cytokine interleukin 10 (IL-10) and anti-inflammatory RAS components was previously observed for AT2 receptors in renal cells [57], and Ang 1-7 in vascular cells [59]. In the present study, we showed that the effect of Ang 1-7 on inhibiting microglial mitochondria fragmentation is mediated by IL-10, specifically by decreasing the activated post-transcriptional phosphorylated Drp1form.

Interestingly, previous studies have observed that some anti-inflammatory effects of IL-10 are mediated by the transcription factor STAT3 in a variety of immune cell types [75, 76]. STAT3 is a transcription factor that regulates several biological functions at the nuclear transcription level, particularly the immune response and inflammation. Activation of STAT3 can play anti- or pro-inflammatory actions depending on cell stimulus. In the case of stimulation by pro-inflammatory cytokines such as IL-6 or IFNγ, STAT3 is tyrosine phosphorylated, dimerized, and translocated to the nucleus to induce specific pro-inflammatory gene expression [77]. However, the IL-10/STAT3 pathway represses the expression of proinflammatory genes at the nuclear transcription level [78, 79]. Interestingly, in addition to the canonical effects at nuclear transcription levels, STAT3 can exert non-canonical functions including mitochondria modulation, microtubule regulation, and heterochromatin stabilization, without tyrosine phosphorylation and DNA-binding [80]. STAT3 can translocate to mitochondria, where it is involved in several mitochondrial functions, such as mitochondrial respiration, mPTP (mitochondrial permeability transition pore) opening, regulation of mitochondrial gene expression or cellular redox homeostasis [50]. However, the mechanisms involved in the translocation of STAT3 to mitochondria remain unknown. In the present study, we showed that the effect of Ang 1-7 on mitochondrial function is through translocation of STAT3 to mitochondria. Under basal conditions, we observed a low but constitutive expression of STAT3 at mitochondria, although pS727-STAT3 was almost absent. However, after Ang 1-7 treatment, both unphosphorylated and Ser727 phosphorylated STAT3, increased notably at mitochondrial levels. Moreover, we showed that the Ang 1-7 effects on STAT3 Ser727 phosphorylation and translocation to mitochondria were mediated by IL-10 signalling. As Ser727 phosphorylated STAT3 was observed to be the effector responsible for mitochondrial functional changes [53], and we observed a constitutive expression and translocation of unphosphorylated STAT3 after Ang 1-7 treatment, we investigated whether Ang 1-7 could mediate phosphorylation of STAT3 inside the mitochondria. Using isolated mitochondria, we demonstrated that Ang 1-7, acting on mitochondrial Ang 1-7 receptors (i.e. Mas and MrgE), produces an increase in the phosphorylated form of STAT3 at Ser727 relative to unphosphorylated STAT3, and that this is mediated by mitochondrial protein kinase A (PKA). Moreover, we showed that Ang 1-7, acting on mitochondrial Ang 1-7 receptors (i.e. Mas and MrgE receptors), produced an increase in intramitochondrial PKA activity, which is responsible for the intramitochondrial phosphorylation of STAT3 at Serine 727.

This role of intramitochondrial PKA is supported by several previous studies showing different effects of intramitochondrial PKA on mitochondrial function through phosphorylation of different targets such as OXPHOS proteins, apoptosis- and dynamic-related proteins [53, 55, 56]. Mitochondrial-located PKA/cAMPc regulates mitochondrial respiration directly by phosphorylation of components of the ETC or indirectly by modulation of mitochondrial proteases [53]. Interestingly, mitochondrial cannabinoid receptors (mtCB1) inhibit PKA-dependent phosphorylation of ETC subunits resulting in decreased cellular respiration [54]. A link between intramitochondrial PKA and major components involved in the effects of Ang 1-7 on mitochondrial dynamics has not previously been shown. However, previous observations on the effects of cellular PKA support our results. Thus, the administration of Ang 1-7 activated cell PKA in cardiomyocytes [81] and glomerular mesangial cells [82]. Furthermore, the main role of the cAMP/PKA pathway in the phosphorylation of STAT3 was also observed in glioma and macrophage cells [83, 84].

In conclusion, the present study shows that the RAS regulates the mitochondrial function in dopaminergic neurons and microglial cells through mitochondrial dynamic regulation and at the respiratory level. The anti-inflammatory effects of Ang 1-7 are related to its role in counteracting the inflammation-induced mitochondrial changes, acting through IL-10 and the STAT3 pathway. Furthermore, we showed the role of mitochondrial Ang 1-7 receptors on increasing mitochondrial phosphorylation of STAT3 *via* increased mitochondrial PKA activity, which is responsible for Ang 1-7 effects at the mitochondrial level. The dysfunction in mitochondrial dynamics is associated with several neurological disorders. Perturbations in mitochondrial dynamics are involved in human neurodegenerative diseases, including Alzheimer's, Parkinson's, Huntington's, and amyotrophic lateral sclerosis [85]. Moreover, two important neuropathies, dominant optic atrophy and Charcot-Marie-Tooth disease, have been directly associated with mutations in genes involved in mitochondrial dynamics. The study of these genetic diseases shows that mitochondrial dynamics alteration is an early pathological event and positions these processes as a major target disease progression modifying therapies [14]. The results of the present study lead to a better understanding of the effects of RAS dysfunction in aging-related diseases, particularly dopaminergic degeneration, and neuroinflammation in Parkinson’s disease.

## Supplementary Materials

The Supplementary data can be found online at: www.aginganddisease.org/EN/10.14336/AD.2024.0981.
