# Supplementary material for: Modulation of Mitochondrial Dynamics by the Angiotensin System in Dopaminergic Neurons and Microglia
Source: Aging Dis. 2024 Oct 22;16(5):3180–203. doi: 10.14336/AD.2024.0981 (PMC12339132; doi:10.14336/AD.2024.0981)
Supplement: Supplementary file 1 [file AD-16-5-3180-s.pdf]

SUPPLEMENTARY DATA

Modulation of Mitochondrial Dynamics by the Angiotensin System in Dopaminergic Neurons and Microglia

Aloia Quijano, Ana I. Rodriguez-Perez, María Alicia Costa-Besada, Andrea Lopez-Lopez, María J. Guerra, Jose Luis Labandeira-Garcia, Rita Valenzuela

© 2024. Quijano A et al. Published online at http://www.aginganddisease.org/EN/10.14336/AD.2024.0981

SUPPLEMENTARY DATA

IMMUNOFLUORESCENCE NEGATIVE CONTROL

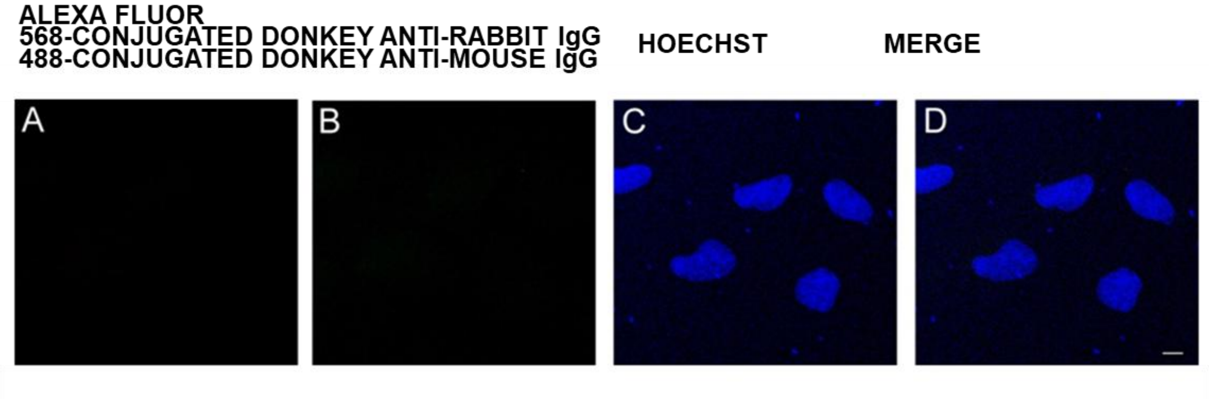

Supplementary Fig. 1. Immunofluorescence of HCM3 cells negative control. (A) The fluorescent 568 nm signal and **(B)** 488 nm signal did not appear in HCM3 cell incubated only with the secondary antibodies (i.e. Alexa Fluor 568-conjugated donkey anti-rabbit IgG and Alexa Fluor 488-conjugated donkey anti-mouse IgG) in the absence of primary antibodies. **(C)** Nuclear staining with Hoechst and **(D)** merged image. Scale bars: 7.5 µm.

© 2024. Quijano A et al. Published online at http://www.aginganddisease.org/EN/10.14336/AD.2024.0981
